# Understanding HIV/AIDS dynamics: insights from CD4+T cells, antiretroviral treatment, and country-specific analysis

**DOI:** 10.3389/fpubh.2024.1324858

**Published:** 2024-04-11

**Authors:** Dipo Aldila, Ranandha P. Dhanendra, Sarbaz H. A. Khoshnaw, Juni Wijayanti Puspita, Putri Zahra Kamalia, Muhammad Shahzad

**Affiliations:** ^1^Department of Mathematics, Universitas Indonesia, Depok, Indonesia; ^2^Department of Mathematics, University of Raparin, Ranya, Iraq; ^3^Department of Mathematics, Universitas Tadulako, Palu, Indonesia; ^4^Department of Mathematics and Statistics, The University of Haripur, Haripur, KP, Pakistan

**Keywords:** HIV/AIDS, antiretroviral treatment, condom, global stability, sensitivity analysis, optimal control, cost-effectiveness

## Abstract

In this article, we present a mathematical model for human immunodeficiency virus (HIV)/Acquired immune deficiency syndrome (AIDS), taking into account the number of CD4+T cells and antiretroviral treatment. This model is developed based on the susceptible, infected, treated, AIDS (SITA) framework, wherein the infected and treated compartments are divided based on the number of CD4+T cells. Additionally, we consider the possibility of treatment failure, which can exacerbate the condition of the treated individual. Initially, we analyze a simplified HIV/AIDS model without differentiation between the infected and treated classes. Our findings reveal that the global stability of the HIV/AIDS-free equilibrium point is contingent upon the basic reproduction number being less than one. Furthermore, a bifurcation analysis demonstrates that our simplified model consistently exhibits a transcritical bifurcation at a reproduction number equal to one. In the complete model, we elucidate how the control reproduction number determines the stability of the HIV/AIDS-free equilibrium point. To align our model with the empirical data, we estimate its parameters using prevalence data from the top four countries affected by HIV/AIDS, namely, Eswatini, Lesotho, Botswana, and South Africa. We employ numerical simulations and conduct elasticity and sensitivity analyses to examine how our model parameters influence the control reproduction number and the dynamics of each model compartment. Our findings reveal that each country displays distinct sensitivities to the model parameters, implying the need for tailored strategies depending on the target country. Autonomous simulations highlight the potential of case detection and condom use in reducing HIV/AIDS prevalence. Furthermore, we identify that the quality of condoms plays a crucial role: with higher quality condoms, a smaller proportion of infected individuals need to use them for the potential eradication of HIV/AIDS from the population. In our optimal control simulations, we assess population behavior when control interventions are treated as time-dependent variables. Our analysis demonstrates that a combination of condom use and case detection, as time-dependent variables, can significantly curtail the spread of HIV while maintaining an optimal cost of intervention. Moreover, our cost-effectiveness analysis indicates that the condom use intervention alone emerges as the most cost-effective strategy, followed by a combination of case detection and condom use, and finally, case detection as a standalone strategy.

## 1 Introduction

Human immunodeficiency virus (HIV) is a virus that infects CD4+ T lymphocytes, leading to a weakened immune system in individuals. On the other hand, Acquired immune deficiency syndrome (AIDS) refers to the symptoms that occur as a result of a weakened immune system due to HIV infection (1). HIV can be transmitted through bodily fluids such as blood, semen, genital fluids, and breast milk. According to data from the WHO and the United Nations Programme on HIV/AIDS (UNAIDS), in 2016, there were 36.7 million people living with HIV (PLHIV)/AIDS worldwide. The majority of people living with HIV are in low and middle-income countries, such as Eswatini, where the prevalence is 25.9% among the adult population, Lesotho with a prevalence of 19.3%, South Africa with a prevalence of 17.8%, Botswana with a prevalence of 16.4%, and Mozambique with a prevalence of 11.6% (2). According to the same source, the top 10 countries with the highest HIV prevalence include 10 African nations, with South Africa having the highest number of people living with HIV, surpassing 7.6 million in 2020. Beyond Africa, the spread of HIV is also a significant concern, particularly in Indonesia, where the latest data report 519,158 individuals affected by HIV as of June 2022 (3). This places Indonesia as the third-highest country with people living with HIV in Asia, following India and Thailand.

The HIV illness may be divided into four phases. People living with HIV (PLHIV) and have entered stage one may experience mild symptoms such as flu-like symptoms, diarrhea, and fever. Those who have entered stage two may experience symptoms such as Tuberculosis (TB), swollen lymph nodes, and skin disorders. In stage three, individuals may experience symptoms in the mucous membranes, such as Tuberculosis (TB) in the lymph nodes. Finally, in stage four, individuals may experience systemic meningoencephalitis. Stage four is commonly referred to as AIDS. HIV attacks CD4+ T cells in the bodies of infected individuals. CD4+ T cells play a crucial role in the immune system and perform many functions in immune activation, coordination, modulation, and regulation (4). AIDS is defined as an outcome among those living with HIV if the CD4+ T cell count is < 200.

Antiretroviral therapy (ART) is available as a treatment to reduce the risk of HIV transmission and lower the amount of HIV in the blood. Highly active antiretroviral therapy (HAART) has been successful in reducing morbidity and mortality among individuals infected with HIV (5). According to the United Nations Programme on HIV/AIDS (UNAIDS),^1^ there were 1.3 million people newly infected with HIV in 2022. Among 39 million people living with HIV in 2022, 630,000 people died of AIDS. According to the global HIV and AIDS epidemic,^2^ only 86% of people with HIV globally knew their HIV status in 2022, the rest of them do not know that they were living with HIV. Therefore, antiretroviral therapy has the potential to reduce the number of individuals infected with HIV in sub-Saharan Africa and other countries.

The mathematical models have been used widely to model the spread of diseases, such as dengue (6), malaria (7, 8), tuberculosis (9–11), HIV (12), COVID-19 (13–16), pneumonia (17). In the context of the HIV/AIDS infectious disease spread, the mathematical model can help researchers understand the impact of interventions and can be used to predict the potential outcome of scenarios in the field. An early mathematical model for HIV/AIDS can be found in Rahman et al.'s study (18). In 2009, Mukandavire et al. (19) presents a mathematical model for HIV/AIDS transmission dynamics, incorporating an explicit incubation period. It demonstrates that effective public health educational campaigns, when targeted at both sexually immature and sexually mature individuals, can significantly slow down the epidemic. The study also identifies the presence of backward bifurcation in the mathematical model, highlighting the complexity of disease dynamics and the potential impact of comprehensive intervention strategies. The presence of the backward bifurcation phenomenon in their model suggests that the extinction of HIV may not solely depend on the condition of the reproduction number being less than one. This is because another endemic possibility may emerge even when the reproduction number is already less than one. In lay terms, backward bifurcation in the model means that even if the conditions initially seem favorable for controlling and reducing HIV (for example, when the reproduction number is less than one), there is still a risk of a resurgence or a persistent presence of the infection. This phenomenon introduces a level of complexity, suggesting that the effectiveness of interventions and the possibility of HIV extinction are not solely determined by one factor (such as a low reproduction number). Instead, additional factors or conditions may influence the dynamics of the infection, making it more challenging to predict and control. Nyabadza and Mukandavire in (20) employ ordinary differential equations to investigate the dynamics of HIV/AIDS, particularly in the context of HIV testing and screening campaigns. The key findings include that having a basic reproduction number below one is necessary but not sufficient for disease control due to backward bifurcation phenomena. Additionally, the study fits the model to real data on HIV prevalence in South Africa, adding empirical validation to the model's insights that HIV counseling and testing itself has very little impact in reducing the prevalence of HIV unless the efficacy of the campaigns exceeds an evaluated threshold in the absence of backward bifurcation. Recently, Zhai et al. (21) develop a stochastic HIV/AIDS model that considers individuals with protection awareness. Their research revealed that HIV can become extinct when the $R_{0}^{s}$ is <1. Furthermore, the study highlights that enhancing the protection efficiency of individuals with awareness and the implementation of continuous antiretroviral therapy (ART) both contribute to reducing the number of people living with HIV (PLHIV), offering potential strategies for disease control. In Jamil et al.'s study (22), a fractal fractional HIV/AIDS model is introduced, using fractional order differential equations. The study utilizes the first and second derivatives of the Lyapunov function to conduct a global analysis of the model. The research suggests that measures to reduce the effective contact rate between susceptible and infected individuals, coupled with improved treatment for those who are already infected, can enhance the effectiveness of interventions against HIV/AIDS. Recently, due to the COVID-19 pandemic, many mathematical models have been introduced to understand the impact of co-infection between HIV/AIDS with COVID-19. Xu et al. (23) focus on developing a mathematical model for HIV-TB co-infection dynamics and validate it using real incidence data from different regions. The article also delves into the comparison of numerical schemes to determine the most effective computational approach for simulating the model. Pinto et al. (24) explore models for HIV and TB coinfection dynamics, considering both fractional and integer order models. Their analysis encompasses treatment strategies for both diseases and includes considerations for the vertical transmission of HIV. Ringa et al. (25) conduct an analysis on sub-models (and co-infection model) related to HIV and COVID-19 co-infection. They apply an optimal control approach to control variables, finding that preventive measures can substantially reduce the burden of co-infections with COVID-19, and effective treatment of COVID-19 could, in turn, reduce co-infections with opportunistic infections such as HIV/AIDS. Please see Omami et al.'s study (26) for another model which incorporates a coinvection between HIV, dengue, and COVID-19. Another classical model was presented by Garba et al. (27), wherein they employed a mathematical framework to simulate the spread of HIV in Nigeria. Their model incorporates factors such as condom use and asymptomatic cases. The researchers utilized incidence data from Nigeria to calibrate the parameters of their model. Readers may refer to (28–34) for more HIV/AIDS related models.

This research is an extension of the study conducted by Rahman et al. (18) with modifications that include the addition of an AIDS compartment as a variable, taking into consideration the population engaged in unprotected sexual intercourse, and the intervention of antiretroviral therapy. This research aims to develop a comprehensive model for the spread of HIV, integrating the dynamics of CD4+T cells and considering key interventions such as condom use and treatment. By fitting model parameters based on data from four top countries with high HIV incidence rates, we seek to provide a nuanced understanding of how these factors influence the trajectory of the epidemic. Additionally, sensitivity analysis was conducted to assess the impact of various model parameters on the number of cases in each country, offering insights into the relative importance of different factors. Furthermore, optimal control techniques are employed to forecast potential optimal scenarios in the field, aiding in the design of effective strategies for HIV prevention and management.

In this study, a mathematical model for the spread of HIV/AIDS through unprotected sexual intercourse has been constructed based on the classification of the number of CD4+ T cells in the body, incorporating the intervention of antiretroviral therapy. The number of CD4+ T cells is crucial in constructing an HIV mathematical model because these cells play a central role in the immune system, and their depletion is a hallmark of HIV infection. CD4+ T cells are a type of white blood cell that helps coordinate the immune response to infections. HIV specifically targets and infects these cells, leading to a decline in their numbers over time. Incorporating the CD4+ T cell count into the mathematical model allows for a more realistic representation of the dynamics of HIV infection and disease progression. Applying some mathematical analysis tools, we conduct an analytical study on our model, including an analysis of existence, the stability analysis of equilibrium points, and the analysis of the basic reproduction number *ℛ*_0_. With this study, we can understand the long-term behavior of HIV transmission as it changes over time. The analysis of the basic reproduction number can quantify which factors play a significant role in efforts to control the spread of HIV. Following that, we conducted numerical simulations, which involved analyzing the elasticity and sensitivity of *ℛ*_0_, in addition to performing autonomous and optimal control simulations using the constructed model. The goal is to gain insights into the impact of antiretroviral therapy on the transmission of HIV/AIDS through unprotected sexual intercourse. This analysis is based on the classification of the number of CD4+ T cells in the body. By interpreting the outcomes from both the analytical study and numerical simulations, we aim to better understand the dynamics and effects of the treatment and condom use on the spread of the virus.

The article is structured as follows: Section 2 presents the construction and analysis of a simple case model where the number of CD4+T cells is not considered in the model. This section includes a global stability of equilibrium points and a sensitivity analysis of the basic reproduction number. Section 3 delves into the mathematical model analysis of the complete model, discussing equilibrium points, control reproduction number, data fitting, and model sensitivity analysis. Section 4 describes the modification of the complete model into a control optimal model. This section includes numerical experiments for different strategies, as well as a cost-effectiveness analysis. Finally, Section 5 provides conclusion.

## 2 A mathematical model of HIV/AIDS with antiretroviral treatment without considering the number of CD4+T cell number class

### 2.1 Model construction

**Figure 1:**
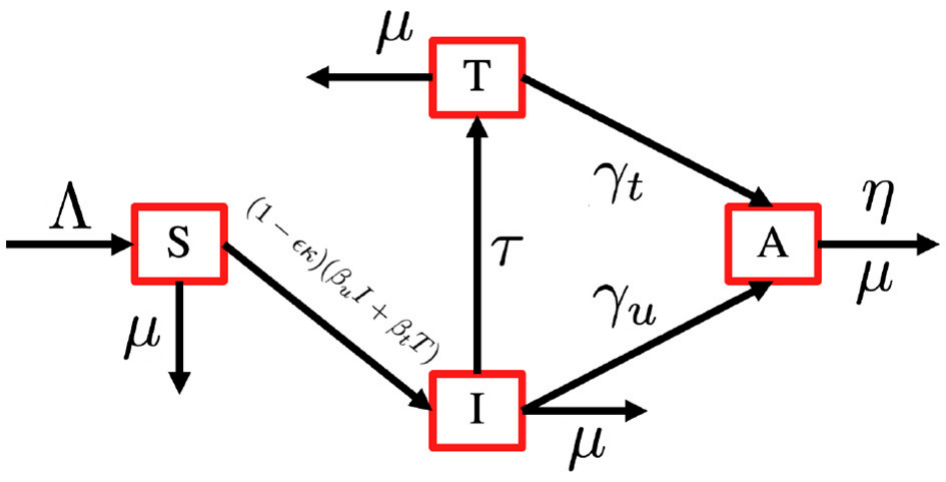
HIV transmission diagram with antiretroviral treatment.

In this section, we consider an antiretroviral treatment for an HIV-infected individual. First, let us consider the total human population (aged 15–49 years), denoted by *N*, to be be categorized into four different compartments based on their health statuses, namely, the susceptible individual (*S*), PLHIV with infection only (*I*), PLHIV receiving treatment (*T*), and PLHIV with AIDS illness (*A*). In this model, we assume that without any test, the HIV-infected individual cannot be detected. Hence, they cannot get a proper treatment. To construct the model, we use the transmission diagram as shown in Figure 1.

The construction of the model is as follows. The recruitment rate of *N* is always considered as a susceptible person from a younger age class of <15 years, with a constant rate Λ. Infection only occurs due to successful contact between susceptible individuals with infected individuals *I* and *T* with a probability of *β*_*u*_ and *β*_*t*_, respectively. The most effective way to prevent sexual transmission of HIV is through abstinence, that is, avoiding all vaginal, anal, and oral sex. In our model, we include the use of a male condom or a female condom that can avoid the transmission of HIV. The variables *ϵ* and *κ* are denoted as condom efficacy and the proportion of people who use condoms, then the transmission rate *β*_*u*_ and *β*_*t*_ can be reduced by a factor of 1 − *ϵκ*. With this assumption, we have *κ* ∈ [0, 1] and *ϵ* ∈ [0, 1]. A bigger value of *κ* represents a better quality of a condom, and a bigger value of *ϵ* represents a condition where more people use condoms during their sexual activity. With the mass action infection process, then the infection force of infection is given by (1 − *ϵκ*)(*β*_*u*_*I* + *β*_*t*_*T*). In this model, we have a transition from *I* to *T* due to a medical test for HIV detection, which is denoted by *τ*. Furthermore, the transition from the HIV stage to AIDS is given by *γ*_*u*_ and *γ*_*t*_ for *I* and *T* individuals, respectively. Due to antiretroviral treatment, we assume that *γ*_*t*_ < *γ*_*u*_. Each compartment has a natural death rate denoted by *μ*, except for *A* compartment which has an additional death rate due to AIDS, which is denoted by η. Based on this assumption, the complete model of HIV/AIDS transmission with the usage of condoms and the intervention of antiretroviral treatment is given by the following system of ordinary differential equations.


(1) $$\begin{array}{l} \begin{array}{l} \frac{dS}{dt}=\Lambda -\left(\left(1-\epsilon \kappa \right)\left(\beta_{u}I+\beta_{t}T\right)+\mu \right)S,\\ \frac{dI}{dt}=\left(1-\epsilon \kappa \right)\left(\beta_{u}I+\beta_{t}T\right)S-\left(\tau +\gamma_{u}+\mu \right)I,\\ \frac{dT}{dt}=\tau I-\left(\gamma_{t}+\mu \right)T,\\ \frac{dA}{dt}=\gamma_{u}I+\gamma_{t}T-\left(\mu +\eta \right)A, \end{array} \end{array}$$

completed with the following initial condition


S(0)>0,I(0)≥0,T(0)≥0,A(0)≥0.

### 2.2 Preliminary analysis

In this section, we describe two theorems to guarantee the positiveness of the solution and also the positive invariant region of the system (1).

** Theorem 1**. All solutions of the HIV/AIDS model in Equation (1) with a non-negative initial condition in ${\mathbb{R}}_{+}^{4}$ remain positive for all time *t* > 0.

*Proof*: From the first equation on the (1), we have


$$\begin{array}{l} \frac{dS}{dt}=\Lambda -\left(\left(1-\epsilon \kappa \right)\left(\beta_{u}I+\beta_{t}T\right)+\mu \right)S\\ \;>-\left(\left(1-\epsilon \kappa \right)\left(\beta_{u}I+\beta_{t}T\right)+\mu \right)S. \end{array}$$

The solution of *S*(*t*) is given by


$$S\left(t\right)>S\left(0\right)\exp\left[-\left(\int_{0}^{t}\left(1-\epsilon \kappa \right)\left(\beta_{u}I\left(\phi \right)+\beta_{t}T\left(\phi \right)\right)+\mu t\right)\right].$$

Since *S*(0) > 0, then *S*(*t*) is always positive for all *t* > 0. The remaining variables *I*(*t*), *T*(*t*), and *A*(*t*) can be shown in a similar way. Hence the solution set {*S, I, T, A*} is always non-negative for all time *t* > 0.    □

** Theorem 2**. The region


$$D=\left\{\left(S,I,T,A\right)\in {\mathbb{R}}_{+}^{4}:0<S+I+T+A\leq \max\left(\frac{\Lambda }{\mu },N\left(0\right)\right)\right\}$$

is positively invariant and attracting with respect to the system (1) with a non-negative initial condition in ${\mathbb{R}}_{+}^{4}$.

*Proof*: Adding all equations in the system (1), we have


$$\frac{dN}{dt}=\Lambda -\mu N-\eta A\leq \Lambda -\mu N.$$

Solving this differential equation with respect to *N*(*t*), we have


$$N\left(t\right)\leq N\left(0\right)\exp\left(-\mu t\right)+\frac{\Lambda }{\mu }\left(1-\exp\left(-\mu t\right)\right).$$

Therefore, we can see that $N\left(t\right)\to \frac{\Lambda }{\mu }$ for *t* → ∞. If $N\left(0\right)>\frac{\Lambda }{\mu }$, then *N*(*t*) will monotonically decrease and tends to $\frac{\Lambda }{\mu }$. If $N\left(0\right)<\frac{\Lambda }{\mu }$, then *N*(*t*) will monotonically increase and tends to $\frac{\Lambda }{\mu }$. On the other hand, if $N\left(0\right)=\frac{\Lambda }{\mu }$, then *N*(*t*) will remain constant for all time *t*, where $N\left(t\right)=\frac{\Lambda }{\mu }$. Hence, according to Theorem 1 and previous calculation, we have $0<S+I+T+A\leq \max\left\{\frac{\Lambda }{\mu },N\left(0\right)\right\}$.    □

Based on Theorems 1 and 2, it is sufficient to consider the dynamics of the system (1) in the region D where the existence, uniqueness, and positiveness of the solution hold.

### 2.3 The HIV/AIDS-free equilibrium point and the basic reproduction number of the model (1)

In this section, we analyze the existence and stability of the HIV/AIDS-free equilibrium points, and how they relate to the basic reproduction number of the system (1). The HIV/AIDS-free equilibrium point of the system (1) is given by


$$\begin{array}{l} E_{1}=\left(S^{0},I^{0},T^{0},A^{0}\right)=\left(\frac{\Lambda }{\mu },0,0,0\right). \end{array}$$

Before we calculate the local and global stability criteria of the HIV/AIDS-free equilibrium points, we first calculate the basic reproduction number of the system (1). First, we decompose the Jacobian matrix of an infected subsystem of the system (1) which is evaluated in $E_{0}$ in a transition **Σ** and transmission *T* matrix as follows:


$$\begin{array}{r} \text{T}=\left[\begin{array}{cc} \frac{\left(1-\epsilon \kappa \right)\beta_{u}\Lambda }{\mu } & \frac{\left(1-\epsilon \kappa \right)\beta_{t}\Lambda }{\mu }\\ 0 & 0 \end{array}\right],\\ \Sigma =\left[\begin{array}{cc} -\tau -\gamma_{u}-\mu & 0\\ \tau & -\gamma_{t}-\mu \end{array}\right]. \end{array}$$

Since the elements of the second row of T are all zero, then defining *E* = [10]^*t*^, the next-generation matrix is given by


$$\begin{array}{l} \begin{array}{l} \text{K}=-{\text{E}}^{t}\text{T}\Sigma^{-1}\text{E}=\left[\frac{\Lambda \left(\beta_{u}\left(\gamma_{t}+\mu \right)+\beta_{t}\tau \right)\left(1-\epsilon \kappa \right)}{\mu \left(\tau +\gamma_{u}+\mu \right)\left(\gamma_{t}+\mu \right)}\right]. \end{array} \end{array}$$

The basic reproduction number of the system (1) is taken by the spectral radius of **K**, which is given by


$$\begin{array}{l} R_{0}=\frac{\Lambda \left(\beta_{u}\left(\gamma_{t}+\mu \right)+\beta_{t}\tau \right)\left(1-\epsilon \kappa \right)}{\mu \left(\tau +\gamma_{u}+\mu \right)\left(\gamma_{t}+\mu \right)}. \end{array}$$

The basic reproduction number in many epidemiological models play an important role in determining the local and global stability of the equilibrium points of their model. The basic reproduction number in our model represents the total number of secondary cases of HIV caused by one primary case of HIV in a completely virgin population during its infection period. The following theorem gives the local stability criteria of $E_{0}$.

** Theorem 3**. The HIV/AIDS-free equilibrium point $E_{1}$ is locally asymptotically stable if $R_{0}<1$ and unstable if $R_{0}>1$.

*Proof*: The Jacobian matrix of the system (1) evaluated in $E_{0}$ is given by


$$J\left(\epsilon_{0}\right)=\left[\begin{array}{cccc} -\mu & -\frac{\left(1-\epsilon \kappa \right)\beta_{u}\Lambda }{\mu } & -\frac{\left(1-\epsilon \kappa \right)\beta_{t}\Lambda }{\mu } & 0\\ 0 & \frac{\left(1-\epsilon \kappa \right)\beta_{u}\Lambda }{\mu }-\mu -\tau -\gamma_{u} & \frac{\left(1-\epsilon \kappa \right)\beta_{t}\Lambda }{\mu } & 0\\ 0 & \tau & -\mu -\gamma_{t} & 0\\ 0 & \gamma_{u} & \gamma_{t} & -\mu -\eta \end{array}\right],$$

which has two explicit eigenvalues, i.e, λ_1_ = − *μ* and λ_2_ = − (*μ* + η), while the other two eigenvalues are taken by the solution of the following polynomial


$$\begin{array}{c} f\left(\lambda \right)=\mu \lambda^{2}+\left(2\mu +\tau +\gamma_{u}+\gamma_{t}\right)\left(1-R_{1}\right)\lambda +\\ \mu \left(\tau +\gamma_{u}+\mu \right)\left(\gamma_{t}+\mu \right)\left(1-R_{0}\right)=0, \end{array}$$

where $R_{1}=\frac{\Lambda \beta_{u}\left(1-\epsilon \kappa \right)}{\mu \left(2\mu +\tau +\gamma_{u}+\gamma_{t}\right)}$ and $R_{0}=\frac{\Lambda \left(\beta_{u}\left(\gamma_{t}+\mu \right)+\beta_{t}\tau \right)\left(1-\epsilon \kappa \right)}{\mu \left(\tau +\gamma_{u}+\mu \right)\left(\gamma_{t}+\mu \right)}$. Since $R_{1}<R_{0}$, then the other two eigenvalues will have a negative real part if $R_{0}<1$. Therefore, the HIV/AIDS-free equilibrium $E_{0}$ is locally asymptotically stable if $R_{0}<1$.     □

The following Theorem gives the global stability criteria of $E_{0}$.

** Theorem 4**. The HIV/AIDS-free equilibrium $E_{1}$ of the system (1) is globally stable if $R_{0}<1$.

*Proof*: Using a same approach as authors in (35), let us define


$$\begin{array}{l} \begin{array}{l} \frac{dX}{dt}=F\left(X,Z\right),\\ \frac{dZ}{dt}=G\left(X,Z\right),G\left(X,0\right)=0, \end{array} \end{array}$$

where *X* = *S* ∈ ℝ_+_ is the compartment of non-infected individuals, and $Z=\left(I,T,A\right)\in {\mathbb{R}}_{+}^{3}$ is the infected compartments. Let $X^{*}=\left(\frac{\Lambda }{\mu },0\right)$.

From direct calculation, we have


$$\begin{array}{l} F\left(X,0\right)=\left[\Lambda -\mu S\right],\\ G\left(X,Z\right)=AZ-Ĝ\left(X,Z\right), \end{array}$$

where


$$\begin{array}{l} A=D_{Z}G\left(X^{*},0\right)=\\ \;\left[\begin{array}{ccc} \frac{\left(1-\epsilon \kappa \right)\beta_{u}}{\mu }-\tau -\gamma_{u}-\mu & \frac{\left(1-\epsilon \kappa \right)\beta_{t}}{\mu } & 0\\ \tau & -\gamma_{t}-\mu & 0\\ \gamma_{u} & \gamma_{t} & -\mu -\eta \end{array}\right],\\ Ĝ\left(X,Z\right)=\left[\begin{array}{c} {Ĝ}_{1}\left(X,Z\right)\\ {Ĝ}_{2}\left(X,Z\right)\\ {Ĝ}_{3}\left(X,Z\right) \end{array}\right]=\left[\begin{array}{c} \begin{array}{c} \left(1-\epsilon \kappa \right)\left(\beta_{u}I+\beta_{t}T\right)\left(\frac{\Lambda }{\mu }-S\right)\\ 0\\ 0 \end{array} \end{array}\right]. \end{array}$$

Since *I* and *T* are always positive, then it is clear that Ĝ(*X, Z*) ≥ 0. It is also clear that X^*^ = (Λ/*μ*) is globally stable for *F*(*X*, 0). Hence, $E_{0}$ is globally asymptotically stable.    □

### 2.4 HIV/AIDS endemic equilibrium point of the system (1)

The HIV/AIDS endemic equilibrium point of system (1) is given by


$$E_{2}=\left(S^{*},I^{*},T^{*},A^{*}\right),$$

where


$$\begin{array}{l} S^{*}=\frac{\left(\gamma_{t}+\mu \right)\left(\tau +\gamma_{u}+\mu \right)}{\left(\beta_{u}\left(\gamma_{t}+\mu \right)+\beta_{t}\tau \right)\left(1-\epsilon \kappa \right)},\\ \;=\frac{\Lambda }{{ℛ}_{0}\mu },\\ I^{*}=\frac{\Lambda (1-\epsilon \kappa )(\beta_{u}(\mu +\gamma_{t})+\beta_{t}\tau )-\mu (\mu +\gamma_{t})(\mu +\tau +\gamma_{u})}{\left((1-\epsilon \kappa )(\beta_{u}(\mu +\gamma_{t})+\beta_{t}\tau )(\mu +\tau +\gamma_{u}\right)}\\ \;=\left(1-\frac{1}{{ℛ}_{0}}\right)\frac{\Lambda }{\mu +\tau +\gamma_{u}},\\ T^{*}=\frac{\tau }{\mu +\gamma_{t}}\frac{\Lambda (1-\epsilon \kappa )(\beta_{u}(\mu +\gamma_{t})+\beta_{t}\tau )-\mu (\mu +\gamma_{t})(\mu +\tau +\gamma_{u})}{\left((1-\epsilon \kappa )(\beta_{u}(\mu +\gamma_{t})+\beta_{t}\tau )(\mu +\tau +\gamma_{u}\right)}\\ \;=\left(1-\frac{1}{{ℛ}_{0}}\right)\frac{\Lambda \tau }{\left(\mu +\gamma_{t})(\mu +\tau +\gamma_{u}\right)},\\ A^{*}=\frac{\Lambda (1-\epsilon \kappa )(\beta_{u}(\mu +\gamma_{t})+\beta_{t}\tau )-\mu (\mu +\gamma_{t})(\mu +\tau +\gamma_{u})}{\left((1-\epsilon \kappa )(\beta_{u}(\mu +\gamma_{t})+\beta_{t}\tau )(\mu +\tau +\gamma_{u}\right)}\frac{\left(\mu \gamma_{u}+\gamma_{t}(\tau +\gamma_{u}\right)}{\left(\mu +\gamma_{t})(\eta +\mu )\right)}\\ \;=\left(1-\frac{1}{{ℛ}_{0}}\right)\frac{\Lambda \left(\mu \gamma_{u}+\gamma_{t}\left(\tau +\gamma_{u}\right)\right)}{\left(\mu +\gamma_{t})(\mu +\tau +\gamma_{u})(\eta +\mu \right)}. \end{array}$$

From the expression of $E_{2}$, we have the following theorem.

** Theorem 5**. The HIV/AIDS endemic equilibrium $E_{2}$ of the system (1) exist in ${\mathbb{R}}_{+}^{4}$ if $R_{0}>1$.

In the following theorem, we show the non-existence of backward-bifurcation phenomena of the system (1).

** Theorem 6**. The system (1) always exhibits a forward bifurcation phenomenon at $R_{0}=1$.

*Proof*: To analyze the bifurcation phenomena of system (1), we use the well-known Castillo-Song bifurcation theorem (35). Please see (36–38) for more examples of the implementation of this theorem in another epidemiological model. First, let us define *S* = *x*_1_, *I* = *x*_2_, *T* = *x*_3_, and *A* = *x*_4_ and *g*_*i*_ for *i* = 1, ..., 4 represent $\frac{dS}{dt},\frac{dI}{dt},\frac{dT}{dt},$ and $\frac{dA}{dt}$, respectively. Next, we choose *β*_*t*_ as the bifurcation parameter. Solving $R_{0}=1$ with respect to *β*_*t*_, we have


$$\begin{array}{l} \beta_{t}^{*}=\frac{\mu \left(\tau +\gamma_{u}+\mu \right)\left(\gamma_{t}+\mu \right)}{\Lambda \left(1-\epsilon \kappa \right)\tau }-\frac{\beta_{u}\left(\gamma_{t}+\mu \right)}{\tau }. \end{array}$$

Next, we calculate the Jacobian matrix of the system (1) and evaluate it at $\beta_{t}^{*}$ and $E_{0}$, we have


$$\begin{array}{l} A=\left[\begin{array}{cccc} -\mu & -\frac{\left(1-\epsilon \kappa \right)\beta_{u}\Lambda }{\mu } & \frac{\left(\Lambda \left(1-\epsilon \kappa \right)\beta_{u}-\mu^{2}-\mu \tau -\mu \gamma_{u}\right)\left(\mu +\gamma_{t}\right)}{\mu \tau } & 0\\ 0 & \frac{\left(1-\epsilon \kappa \right)\beta_{u}\Lambda }{\mu }-\mu -\tau -\gamma_{u} & -\frac{\left(\Lambda \left(1-\epsilon \kappa \right)\beta_{u}-\mu^{2}-\mu \tau -\mu \gamma_{u}\right)\left(\mu +\gamma_{t}\right)}{\mu \tau } & 0\\ 0 & \tau & -\mu -\gamma_{t} & 0\\ 0 & \gamma_{u} & \gamma_{t} & -\eta -\mu \end{array}\right] \end{array}$$

The eigenvalues of A are


$$\begin{array}{c} \lambda_{1}=0,\lambda_{2}=-\mu ,\lambda_{3}=-\left(\mu +\eta \right),\text{ and }\lambda_{4}=\\ \frac{\Lambda \left(1-\kappa \epsilon \right)\beta_{u}-\mu \left(2\mu +\tau +\gamma_{t}+\gamma_{u}\right)}{\mu }. \end{array}$$

We can see clearly that we have a simple zero eigenvalue, and λ_2_ and λ_3_ are negative. We have λ_4_ < 0 if and only if $\frac{\Lambda \left(1-\kappa \epsilon \right)\beta_{u}}{\mu \left(2\mu +\tau +\gamma_{t}+\gamma_{u}\right)}<1$. Since


$$\begin{array}{c} R_{0}=1=\frac{\Lambda \left(\beta_{u}\left(\gamma_{t}+\mu \right)+\beta_{t}\tau \right)\left(1-\epsilon \kappa \right)}{\mu \left(\tau +\gamma_{u}+\mu \right)\left(\gamma_{t}+\mu \right)}>\frac{c\Lambda \beta_{u}}{\mu \left(\mu +\tau +\gamma_{u}\right)}>\\ \frac{\Lambda \left(1-\kappa \epsilon \right)\beta_{u}}{\mu \left(2\mu +\tau +\gamma_{t}+\gamma_{u}\right)}, \end{array}$$

then we also have λ_4_ < 0. Hence, we can proceed to the next step to analyze the bifurcation type of our model. The bifurcation type of our model can be determined with the following formula:


$$\begin{array}{ll} a=\overset{4}{\underset{k,i,j=1}{\sum }}v_{k}w_{i}w_{j}\frac{\partial^{2}g_{k}}{dx_{i}dx_{j}}\left(\text{0},0\right), & b=\overset{4}{\underset{k,i=1}{\sum }}v_{k}w_{i}\frac{\partial^{2}g_{k}}{dx_{i}d\beta_{t}}\left(\text{0},0\right), \end{array}$$

where *v* and *w* is the left and right eigenvectors of A with respect to the zero eigenvalue, respectively. The left eigenvalue of A with respect to the eigenvalue 0 is given by


$$\begin{array}{r} \begin{array}{r} v_{1}=0,v_{2}=1>0,v_{3}=\\ \frac{\left(\Lambda \left(1-\kappa \epsilon \right)\beta_{u}-\mu \left(\mu +\tau +\gamma_{u}\right)\right)}{\tau \mu }<0,\text{ with }v_{4}=0. \end{array} \end{array}$$

On the other hand, the right eigenvector of A with respect to the eigenvalue 0 is given by


$$\begin{array}{r} \begin{array}{r} w_{1}=-\frac{\left(\gamma_{t}+\mu \right)\left(\tau +\gamma_{u}+\mu \right)}{\mu \tau },w_{2}=\frac{\gamma_{t}+\mu }{\tau },w_{3}=1,\text{ and}\\ w_{4}=\frac{\mu \gamma_{u}+\tau \gamma_{t}+\gamma_{t}\gamma_{u}}{\left(\eta +\mu \right)}. \end{array} \end{array}$$

Hence, we have


$$\begin{array}{l} a=\overset{4}{\underset{k,i,j=1}{\sum }}v_{k}w_{i}w_{j}\frac{\partial^{2}g_{k}}{dx_{i}dx_{j}}\left(\text{0},0\right)\\ \;=-\frac{2\left(\tau +\gamma_{u}+\mu \right)\left(\beta_{t}\tau +\beta_{u}\left(\gamma_{t}+\mu \right)\right)\left(\gamma_{t}+\mu \right)\left(1-\epsilon \kappa \right)}{\tau^{2}\Lambda },\\ b=\overset{4}{\underset{k,i=1}{\sum }}v_{k}w_{i}\frac{\partial^{2}g_{k}}{dx_{i}d\beta_{t}}\left(\text{0},0\right)\\ \;=\left(1-\epsilon \kappa \right). \end{array}$$

Since *a* < 0 and *b* > 0, the system (1) always exhibits a forward bifurcation at $R_{0}=1$.     □

The following corollary is a direct implication from Theorem 6.

** Corollary 1**. The HIV/AIDS endemic equilibrium $E_{2}$ of the system (1) is locally stable for $R_{0}>1$ but close to one.

In epidemiology, bifurcation refers to a qualitative change in the behavior of a dynamical system as a parameter is varied. Forward bifurcation specifically occurs when a stable endemic equilibrium coexists with an unstable disease-free equilibrium at parameter values where the basic reproduction number ($R_{0}$) is larger than one. On the other hand, in cases where the reproduction number falls below one, a stable disease-free equilibrium exists without the existence of the endemic equilibrium. Consequently, the condition where the reproduction number equals one marks the bifurcation point. In the context of the HIV/AIDS model that we developed in Equation (1), forward bifurcation has significant implications. It highlights the importance of not only reducing transmission rates but also addressing factors that contribute to the maintenance of stable disease-free equilibrium, such as the efficacy of condom use and treatment. By considering the implications of forward bifurcation, epidemiologists and policymakers can develop more nuanced and effective approaches to combating the HIV/AIDS epidemic.

### 2.5 Effect of $R_{0}$ to endemic size $E_{2}$

Here in this section, we will perform the elasticity index of the basic reproduction number $R_{0}$ and the endemic equilibrium size of the HIV/AIDS model in Equation (1) and also the parameter sensitivity to $R_{0}$. We use parameter values for Lesotho (See table in Appendix 2), except for *τ* and *κ*, which is given as follows:


(2) $$\begin{array}{c} \beta_{u}=1.4888\times 10^{-6},\beta_{t}=1.14888\times 10^{-7},\mu =\frac{1}{57},\\ \gamma_{u}=0.6,\gamma_{t}=0.3,\tau =0.1,\epsilon =0.925,\kappa =0.1,\text{ and}\\ \Lambda =\frac{736680}{57}. \end{array}$$

With this set of parameters, we have $R_{0}=1.43$, which is larger than one. Hence, the HIV/AIDS endemic equilibrium $E_{2}$ exists and is given by


$$E_{2}=\left(S,I,T,A\right)=\left(514872,5423,1707,15709\right)$$

is locally stable. This confirms the results of Theorems 5 and 6.

Next, we calculate the elasticity index of $R_{0}$ and the endemic equilibrium size. To perform this simulation, we use the following formula (39):


$$\begin{array}{l} E_{Q}^{p}=\frac{\partial Q}{\partial p}\times \frac{p}{Q}, \end{array}$$

where *p* is the model parameter, and *Q* is the quantity of the model output, such as $R_{0}$ or endemic equilibrium size $E_{2}$. Using the above formula, we have


$$\begin{array}{l} E_{R_{0}}^{\beta_{u}}=\frac{\beta_{u}\left(\mu +\gamma_{t}\right)}{\beta_{u}\left(\mu +\gamma_{t}\right)+\tau \beta_{t}}. \end{array}$$

**Figure 2:**
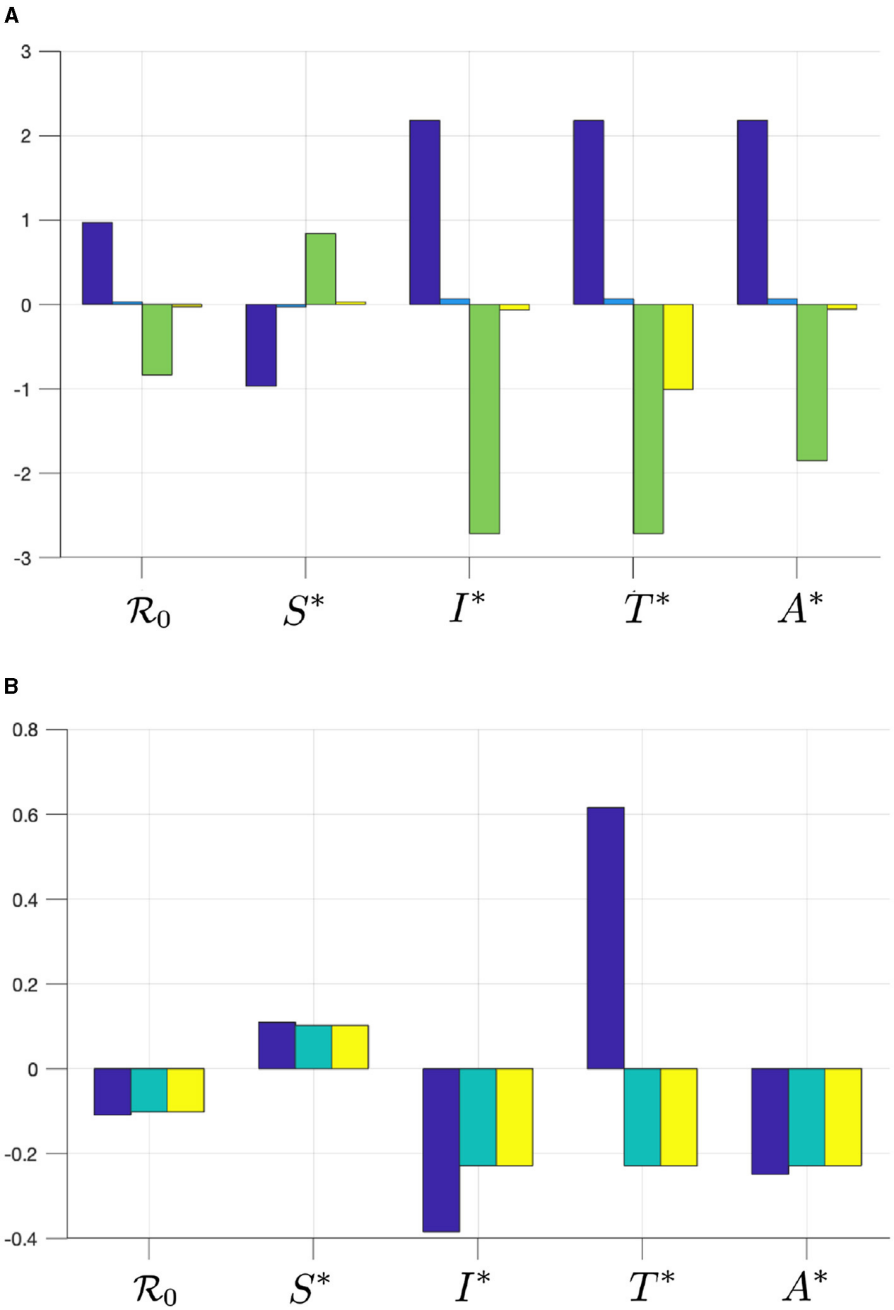
The elasticity of $R_{0}$, S^*^, I^*^, T^*^, and A^*^ with respect to **(A)** *β*_*u*_ (blue), *β*_*t*_ (cyan), *γ*_*u*_ (green), and *γ*_*t*_ (yellow), and with respect to **(B)** *τ* (blue), *κ* (cyan), and *ϵ* (yellow).

Substituting parameter values in Equation (2) yield $E_{R_{0}}^{\beta_{u}}=+0.969$, which means that increasing *β*_*u*_ by 1% will increase $R_{0}$ by 0.969%. Therefore, if we increase *β*_*u*_ from 1.4888 × 10^−7^ to 1.503 × 10^−7^ (increased by 1%), then $R_{0}$ increases from 1.43 to 1.44, which is an increase of 0.969%. With the same approach, we can calculate the elasticity of each parameter in the system (1) (except Λ that we ignored since the number of recruitment rates cannot be changed in the field) with respect to $R_{0}$ and $E_{2}$. The results can be seen in Figure 2.

From Figure 2, we can see that *β*_*u*_ has a significant impact on $R_{0}$ and $E_{0}$, more dominant than *β*_*t*_. Hence, infection from untreated individuals plays a significant role in determining the spread of HIV/AIDS. Increasing *β*_*u*_ and *β*_*t*_ will increase $R_{0}$ and all infected compartments in $E_{0}$, but reduce *S* in $E_{0}$. Furthermore, we can see that the progression to the *A* compartment, which is presented by parameters *γ*_*u*_ and *γ*_*t*_, will reduce $R_{0}$. We can also see that *γ*_*u*_ is more significant in affecting $R_{0}$ or all compartments in $E_{0}$ compared to *γ*_*t*_. Another important result is that the case detection rate *τ* is promising in reducing $R_{0}$ and all infected compartments.

**Figure 3:**
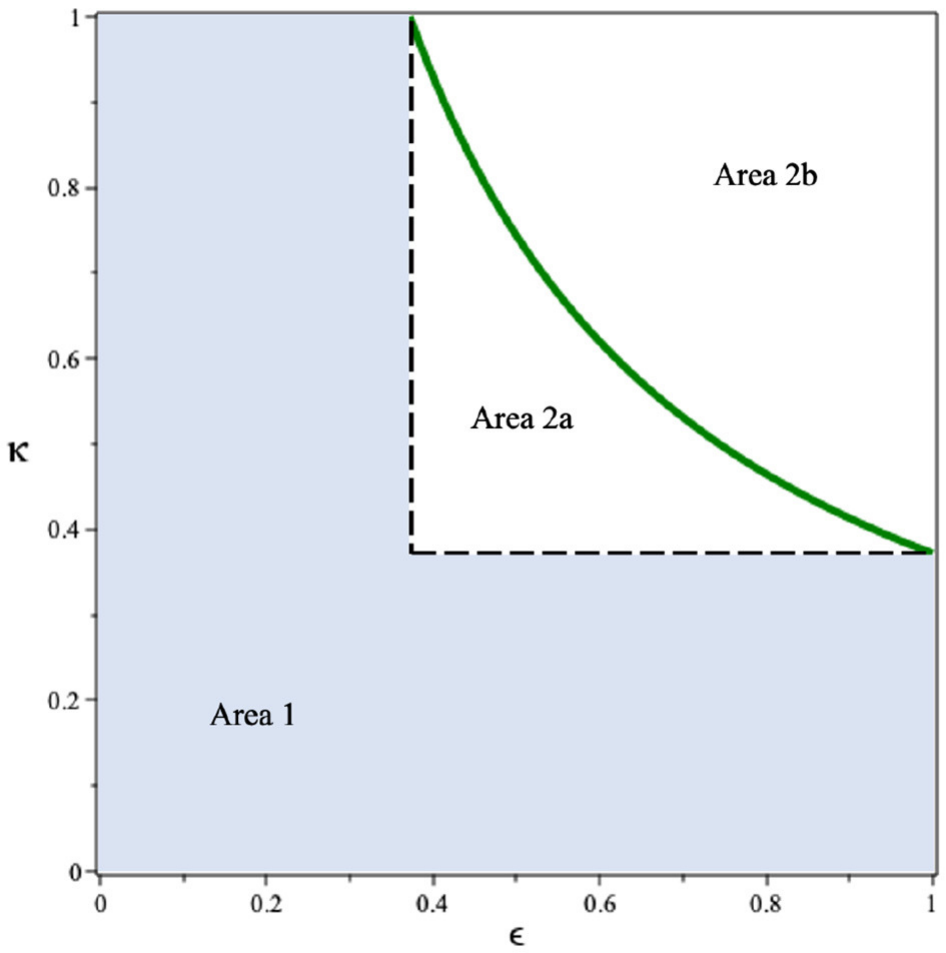
Dependency of $R_{0}$ on the condom efficacy and the proportion of people who use it.

Since we can see that the use of condoms to reduce the infection rates *β*_*u*_ and *β*_*t*_ shows a promising potential to control HIV/AIDS, it is necessary to see the combination of *ϵ* and *κ* to reduce $R_{0}$. By substituting all parameter values in Equation (2), except *ϵ* and *κ*, into $R_{0}$, we have $R_{0}\left(\epsilon ,\kappa \right)=1.591\left(1-\epsilon \kappa \right).$ Drawing this function in the *ϵ*−*κ* plane, we have the results in Figure 3. It can be seen that if the efficacy of the condom or the proportion of people who use condoms is <0.37, then the basic reproduction number will always be larger than one (Area 1). Hence, the endemic situation will always appear in the population. On the other hand, if the two above mentioned parameters are >0.37 (Area 2), then there is a chance to eradicate HIV from the population (Area 2b).

## 3 A mathematical model of HIV/AIDS considering CD4+T cell number

### 3.1 Model construction

In this section, we modify our previous model in the system (1) by accommodating the number of CD4+T cells in the human body. To construct the model, we use the following assumptions:

1. We classify each of the *I* and *T* compartments in the system (1) into three compartments, which present the class of infected and treated individuals based on the number of CD4+T cells. We denote *I*_1_, *I*_2_, and *I*_3_ as infected individuals who have not yet been detected and have different average numbers of CD4+T cells in their blood. Please see the descriptions of *I*_*i*_ and *T*_*i*_ for *i* = 1, 2, 3 in Table 1.
2. We assume that since HIV infection itself is not very harmful, there is no additional death rate attributable directly to HIV individuals (compartments *I*_*i*_ and *T*_*i*_). However, additional deaths occur only among individuals with AIDS (compartment *A*), and this occurs at a constant rate represented by the parameter η.
3. The case detection test can determine the level of CD4+T cells in the human body. Hence, we have a transition rate *τ* from *I*_*i*_ to *T*_*i*_ for *i* = 1, 2, 3 due to successful case detection.
4. Each treated compartment will get antiretroviral treatment which will delay the progression of HIV to AIDS. We assume that successful treatment will increase the number of CD4+T cells. With a duration of treatment is *ρ*, we have the probability of treatment being unsuccessful given by *s, q*, and *r* for individuals in *T*_1_, *T*_2_, and *T*_3_, respectively.
5. The infection rate of individuals in *I*_*i*_ is given by *β*_*u*_ while for individuals in *T*_*i*_ is given by *β*_*t*_. As an effect of treatment, here we assume *β*_*t*_ < *β*_*u*_.

**Table 1:** Description of variables of the HIV/AIDS model in the system (3).

**Variable**	**Description**
*S*	Number of susceptible individuals
*I*_1_/*I*_2_/*I*_3_	Number of people living with HIV who have number of CD4+T cells in the range of > 500/200 − 499/ < 200 cells/mm^3^
*T*_1_/*T*_2_/*T*_3_	Number of people living with HIV and get treated who have number of CD4+T cells in the range of > 500/200 − 499/ < 200 cells/mm^3^
*A*	Number of people living with HIV with AIDS illness

**Figure 4:**
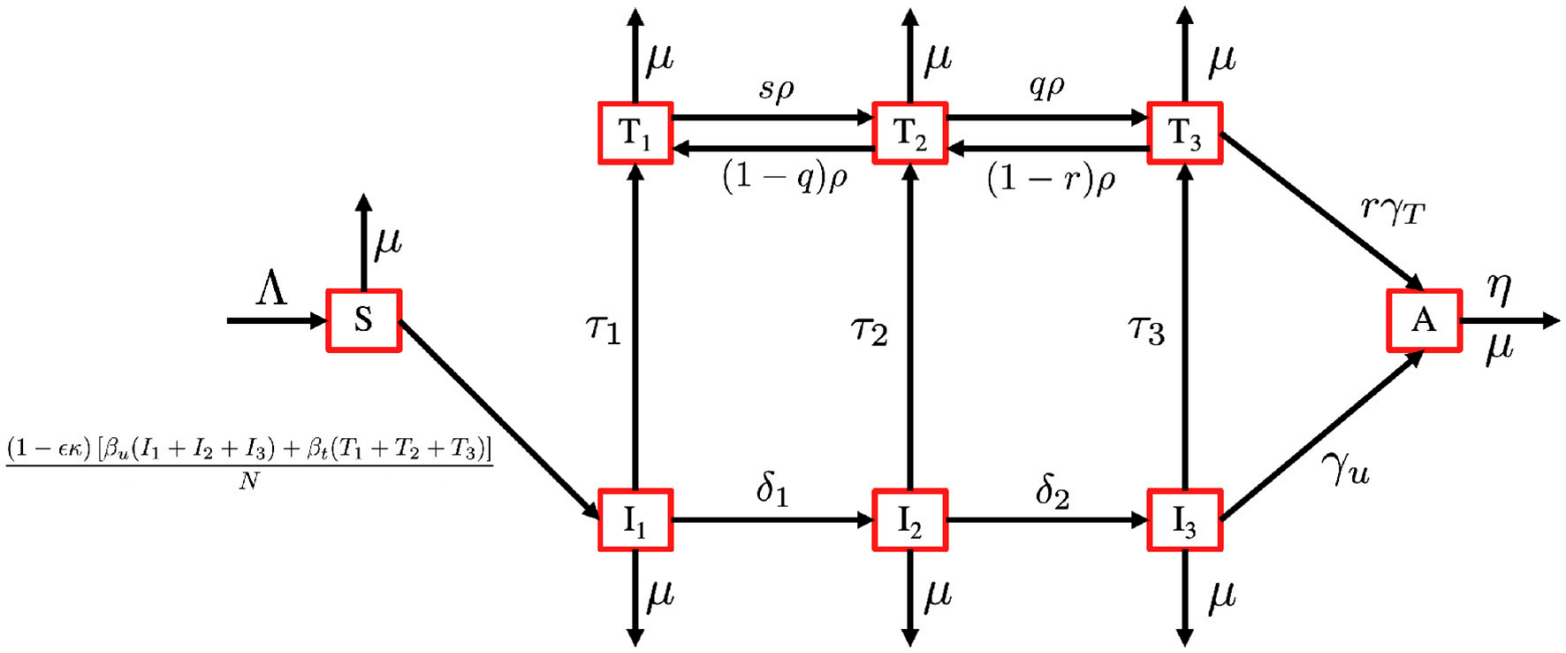
Transmission diagram of the HIV/AIDS model in system (3).

Based on this model description and the transmission diagram in Figure 4, the mathematical model of HIV/AIDS considering the level of CD4+T cells, antiretroviral treatment, and case detection is given by the following system of differential equations.


(3) $$\begin{array}{l} \begin{array}{l} \frac{dS}{dt}=\Lambda -\left(\left(1-\epsilon \kappa \right)\left[\beta_{u}\left(I_{1}+I_{2}+I_{3}\right)+\beta_{t}\left(T_{1}+T_{2}+T_{3}\right)\right]+\mu \right)S,\\ \frac{dI_{1}}{dt}=\left(1-\epsilon \kappa \right)\left[\beta_{u}\left(I_{1}+I_{2}+I_{3}\right)+\beta_{t}\left(T_{1}+T_{2}+T_{3}\right)\right]S-\\ \;\left(\tau_{1}+\delta_{1}+\mu \right)I_{1},\\ \frac{dI_{2}}{dt}=\delta_{1}I_{1}-\left(\tau_{2}+\delta_{2}+\mu \right)I_{2},\\ \frac{dI_{3}}{dt}=\delta_{2}I_{2}-\left(\tau_{3}+\gamma_{u}+\mu \right)I_{3},\\ \frac{dT_{1}}{dt}=\tau_{1}I_{1}+\left(1-q\right)\rho T_{2}-\left(s\rho +\mu \right)T_{1},\\ \frac{dT_{2}}{dt}=\tau_{2}I_{2}+s\rho T_{1}+\left(1-r\right)\rho T_{3}-\left(\left(1-q\right)\rho +q\rho +\mu \right)T_{2},\\ \frac{dT_{3}}{dt}=\tau_{3}I_{3}+q\rho T_{2}-\left(\left(1-r\right)\rho +r\gamma_{t}+\mu \right)T_{3},\\ \frac{dA}{dt}=r\gamma_{t}T_{3}+\gamma_{u}I_{3}-\left(\mu +\eta \right)A, \end{array} \end{array}$$

completed with a non-negative initial condition *S*(0) ⩾ 0, *I*_1_(0) ⩾ 0, *I*_2_(0) ⩾ 0, *I*_3_(0) ⩾ 0, *T*_1_(0) ⩾ 0, *T*_2_(0) ⩾ 0, *T*_3_(0) ⩾ 0, and *A*(0) ⩾ 0.

### 3.2 Preliminary analysis

With a similar approach, as we have given for Theorems 1 and 2, the positivity and boundedness criteria of the system (3) is given in the following theorems.

** Theorem 7**. All solutions of the HIV/AIDS model in Equation (3) with a non-negative initial condition in ${\mathbb{R}}_{+}^{8}$ remain positive for all time *t* > 0.

** Theorem 8**. The region


$$\begin{array}{c} 𝒟=\{(S,I_{1},I_{2},I_{3},T_{1},T_{2},T_{3},A)\in {\mathbb{R}}_{+}^{8}:0<S+I_{1}+I_{2}+I_{3}+T_{1}+\;\\ T_{2}+T_{3}+A\leq \max\left(\frac{\Lambda }{\mu },N(0)\right)\} \end{array}$$

is positively invariant and attracting with respect to the system (1) with a non-negative initial condition in ${\mathbb{R}}_{+}^{8}$.

### 3.3 The HIV/AIDS-free equilibrium and the control reproduction number

The HIV/AIDS-free equilibrium of the system (3) is given by


$$\begin{array}{l} {ℰ}_{1}=\left(S^{0},I_{1}^{0},I_{2}^{0},I_{3}^{0},T_{1}^{0},T_{2}^{0},T_{3}^{0},A^{0}\right)=\left(\frac{\Lambda }{\mu },0,0,0,0,0,0,0\right). \end{array}$$

Using the next-generation method (40), we determine the expression of the basic reproduction number of the system (3). Similar to the previous section, we first determine the transition (Σ) and transmission (**T**) matrices of the infected subcompartment of the system (3), which is given by


$$\begin{array}{c} T=\left[\begin{array}{cccccc} \frac{\Lambda \left(1-\epsilon \kappa \right)\beta_{u}}{\mu } & \frac{\Lambda \left(1-\epsilon \kappa \right)\beta_{u}}{\mu } & \frac{\Lambda \left(1-\epsilon \kappa \right)\beta_{u}}{\mu } & \frac{\Lambda \left(1-\epsilon \kappa \right)\beta_{t}}{\mu } & \frac{\Lambda \left(1-\epsilon \kappa \right)\beta_{t}}{\mu } & \frac{\Lambda \left(1-\epsilon \kappa \right)\beta_{t}}{\mu }\\ 0 & 0 & 0 & 0 & 0 & 0\\ 0 & 0 & 0 & 0 & 0 & 0\\ 0 & 0 & 0 & 0 & 0 & 0\\ 0 & 0 & 0 & 0 & 0 & 0\\ 0 & 0 & 0 & 0 & 0 & 0 \end{array}\right], \end{array}$$

and


$$\begin{array}{l} \Sigma =\left[\begin{array}{cccccc} -a_{1} & 0 & 0 & 0 & 0 & 0\\ \delta_{1} & -a_{2} & 0 & 0 & 0 & 0\\ 0 & \delta_{2} & -a_{3} & 0 & 0 & 0\\ \tau_{1} & 0 & 0 & -a_{4} & \left(1-q\right)\rho & 0\\ 0 & \tau_{2} & 0 & \rho s & -a_{5} & \left(1-r\right)\rho \\ 0 & 0 & \tau_{3} & 0 & q\rho & -a_{6} \end{array}\right], \end{array}$$

where *a*_1_ = *τ*_1_ + δ_1_ + *μ*, *a*_2_ = *τ*_2_ + δ_2_ + *μ*, , *a*_3_ = *τ*_3_ + *γ*_*u*_ + *μ*, *a*_4_ = *ρs* + *μ*, *a*_5_ = (1 − *q*)*ρ* + *q*ρ** + *μ*, and *a*_6_ = (1 − *r*)*ρ* + *r*γ**_*t*_ + *μ*. Since we have only the first row of **T** is non-zero, while the others are zero, we define


$$\begin{array}{l} \text{E}=\left[\begin{array}{c} 1\\ 0\\ 0\\ 0\\ 0\\ 0 \end{array}\right], \end{array}$$

and calculate the control reproduction number using the formula of **K** = − **E**^*t*^**T**Σ^−1^**E**. Hence, the control reproduction number of the system (3) is given by


$$\begin{array}{l} {ℛ}_{0}=\rho \left(\text{K}\right)=\frac{b_{1}}{a_{1}}+\frac{b_{1}\delta_{1}}{a_{1}a_{2}}+\frac{b_{1}\delta_{1}\delta_{2}}{a_{1}a_{2}a_{3}}+b_{2}K_{1}+b_{2}K_{2}+b_{2}K_{3}, \end{array}$$

where


$$\begin{array}{l} \begin{array}{l} b_{1}=\frac{\Lambda \left(1-\epsilon \kappa \right)\beta_{u}}{\mu },\\ b_{2}=\frac{\Lambda \left(1-\epsilon \kappa \right)\beta_{t}}{\mu },\\ K_{1}=\frac{\left(-\left(r-1\right)\left(\left(a_{2}a_{3}+\delta_{1}\delta_{2}\right)q-\delta_{1}\delta_{2}\right)\rho^{2}+a_{6}\delta_{1}a_{3}\left(q-1\right)\rho -a_{2}a_{3}a_{5}a_{6}\right)\tau_{1}}{a_{1}\left(\left(\left(a_{6}s+a_{4}\left(r-1\right)\right)q-a_{6}s\right)\rho^{2}+a_{4}a_{5}a_{6}\right)a_{2}a_{3}},\\ K_{2}=\frac{\tau_{2}\left(\left(-\delta_{1}\delta_{2}\left(r-1\right)a_{4}+a_{6}sa_{2}a_{3}\right)\rho +a_{3}a_{4}a_{6}\delta_{1}\right)}{a_{1}\left(\left(q\left(r-1\right)a_{4}+a_{6}s\left(q-1\right)\right)\rho^{2}+a_{4}a_{5}a_{6}\right)a_{2}a_{3}},\text{ and}\\ K_{3}=\frac{\left(s\left(\left(a_{2}a_{3}+\delta_{1}\delta_{2}\right)q-\delta_{1}\delta_{2}\right)\rho^{2}+q\rho a_{3}a_{4}\delta_{1}+a_{4}a_{5}\delta_{1}\delta_{2}\right)\tau_{3}}{a_{1}\left(\left(\left(a_{6}s+a_{4}\left(r-1\right)\right)q-a_{6}s\right)\rho^{2}+a_{4}a_{5}a_{6}\right)a_{2}a_{3}}. \end{array} \end{array}$$

Following (41) , the local stability criteria of *ℰ*_1_ is given in the following theorem.

** Theorem 9**. The HIV/AIDS-free equilibrium of the system (3) given by *ℰ*_1_ is locally asymptotically stable if *ℛ*_0_ < 1 and unstable if *ℛ*_0_ > 1.

### 3.4 Data fitting

Here in this section, we fit the model (3) to the data of HIV prevalence (age 15–49 years) from Eswatini, Lesotho, Botswana, and South Africa, which represent the top four countries with the highest prevalence of HIV in the world in 2023. The prevalence data that is shown in Appendix 1 can be download from (42). Some parameters on the model in Equation (3) were estimated using the data-fitting process, while the other parameters were taken from the references. Here is the explanation of how we choose the value of these parameters.

1. The total population N, drawn from individuals aged between 15 and 64 years in each country, is sourced from The World Bank data in (43). According to this data, the populations of Eswatini, Lesotho, Botswana, and South Africa are 736,680, 1,425,560, 1,676,630, and 39,264,160, respectively.
2. The natural death rate is denoted by *μ*. Using data from the World Bank (44), we have $\mu^{\text{Eswatini}}=\frac{1}{57}{\text{year}}^{-1},\mu^{\text{Lesotho}}=\frac{1}{53}{\text{year}}^{-1},\mu^{\text{Botswana}}=\frac{1}{61}{\text{year}}^{-1},$ and $\mu^{\text{South Africa}}=\frac{1}{62}{\text{year}}^{-1}$.
3. Recruitment rate is denoted by (Λ). We assume that the total natural death is approximately equal to the total newborn, hence we have Λ = *μN*.
4. Condom efficacy is denoted by (*ϵ*). Based on several references (45), the efficacy of the condom usage is between 90 and 95%. Hence, we assume that *ϵ* = 92.5%.
5. The progression rate due to the decreases in the number of CD4 + T Cells (δ_1_, δ_2_). Based on (18), we choose δ_1_ = 0.33 and δ_2_ = 0.34.
6. Based on (27), we use *γ*_*u*_ = 0.6. Since *γ*_*t*_ < *γ*_*u*_ due to treatment that has been followed by *T* individuals, then we assumed *γ*_*t*_ = 0.3.
7. Since *q, r*, and *s* are proportions, we assume that *ρ* = 1.64, *r* = 0.5, *q* = 0.653, and *s* = 0.653.
8. Due to the treatment undertaken by individuals in *T*, then we assume that *β*_*t*_ < *β*_*u*_.
9. We assume that individuals in *T*_3_ are easier to be asked to follow the treatment process for HIV rather than individuals in *T*_1_ and *T*_2_. Hence, we assume *τ*_3_ > *τ*_2_ > *τ*_1_ > 0.
10. Since *κ* is the proportion of people who use condoms during sexual activity, we assume *κ* ∈ [0, 1].

**Figure 5:**
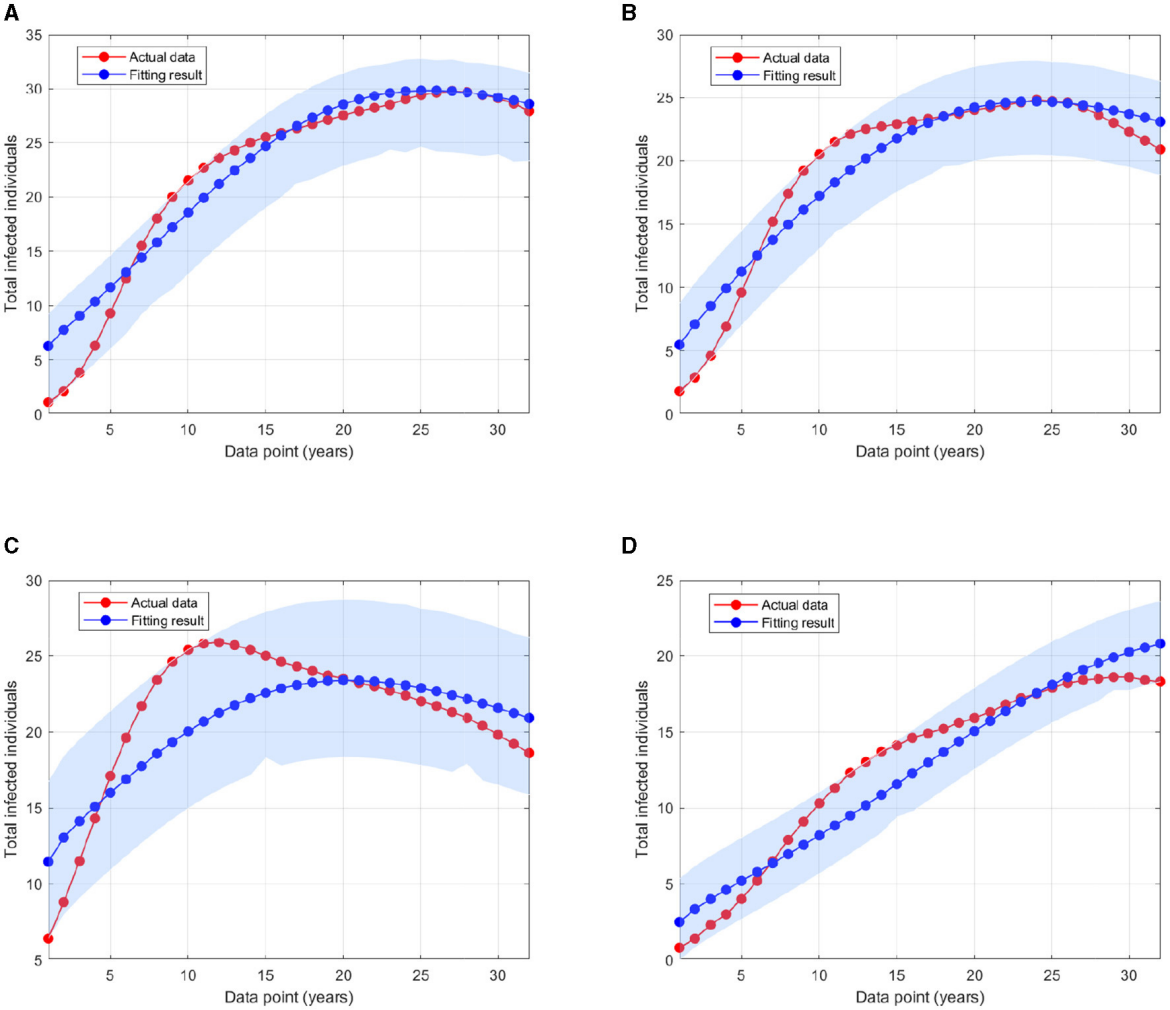
A comparison of HIV prevalence based on actual data and the model obtained from the estimated parameters for **(A)** Eswatini; **(B)** Lesotho; **(C)** Botswana; and **(D)** South Africa.

Our aim is to estimate *β*_*u*_, *β*_*t*_, *τ*_1_, *τ*_2_, *τ*_3_, and *ϵ* which minimize the following error function


$$\begin{array}{l} \text{Error}=\overset{32}{\underset{i=1}{\sum }}{\left(\frac{I_{1}+I_{2}+I_{3}+T_{1}+T_{2}+T_{3}+A}{N}\times 100-P_{i}\right)}^{2}, \end{array}$$

where *P*_*i*_ is the HIV prevalence data at time step − *i* and *N* is the total population in 1990 for Eswatini, Lesotho, Botswana, and South Africa as described before. The initial conditions of *I*_*i*_(0), *T*_*i*_(0), and *A*(0), for *i* = 1, 2, 3 are also estimated here, with $S\left(0\right)=N-\overset{3}{\underset{i=1}{\sum }}\left(I_{i}\left(0\right)+T_{i}\left(0\right)+A\left(0\right)\right)$. The particle swarm optimization (PSO) is used to find the minimum of error function. By using the PSO algorithm as given in (46), with the number of particles given is 500, the maximum iteration is 1,000, and *c*_1_ = *c*_2_ = 1, we obtain the estimation of the parameters and the initial conditions as shown in Appendix 2. Figure 5 displays the weekly prevalence calculations from the estimated results compared to the HIV prevalence data for Eswatini, Lesotho, Botswana, and South Africa. The model simulation in Figure 5 shows a better agreement with the actual data. The basic reproduction numbers for each country are given as follows: 1.095, 1.682, 1.732, and 1.65 for Eswatini, Lesotho, Botswana, and South Africa, respectively. A 95% confidence interval for the fitting results using bootstrap resampling residual approach is also given in Figure 5. The lower and upper bounds of the confidence interval are computed by sorting 1,000 bootstrap samples and taking the 2.5 and 97.5% percentiles.

### 3.5 Sensitivity analysis of the model and the control reproduction number

The sensitivity methods can be used on infectious disease models to determine which variable or parameter is sensitive to a specific condition. Identifying the key critical parameters is an effective way to study such models more widely and accurately. Recently, we have used the sensitivity methods to identify the critical parameters for some infectious disease models. Suppose that an infectious disease model has *m* compartments *x*_*i*_ for *i* = 1, 2, ..., *m* and *n* parameters *k*_*j*_ for *j* = 1, 2, ..., *n*, then, the local sensitivities can be calculated using three different techniques: non-normalizations, half-normalizations, and full-normalizations. First, the equation of non-normalization sensitivities is given by


$$\begin{array}{l} \begin{array}{c} S_{k_{j}}^{x_{i}}=\frac{\partial x_{i}}{\partial k_{j}}, \end{array} \end{array}$$

where $S_{k_{j}}^{x_{i}}$ is measured as a sensitivity coefficient of each *x*_*i*_ with respect to each parameter *k*_*j*_. Then, the formula of half-normalization sensitivities is also defined below:


$$\begin{array}{l} \begin{array}{c} S_{k_{j}}^{x_{i}}=\left(\frac{1}{x_{i}}\right)\left(\frac{\partial x_{i}}{\partial k_{j}}\right). \end{array} \end{array}$$

Finally, the equation of full-normalization sensitivities is defined by


$$\begin{array}{l} \begin{array}{c} S_{k_{j}}^{x_{i}}=\left(\frac{k_{j}}{x_{i}}\right)\left(\frac{\partial x_{i}}{\partial k_{j}}\right). \end{array} \end{array}$$

We have used such estimated parameters and initial variables in computational simulations using MATLAB. The results given in this work provide an important step forward to understand the model dynamics more widely. This helps us to identify critical model parameters and how each model state is affected by the model parameters.

#### 3.5.1 Model sensitivity analysis in Eswatini

**Figure 6:**
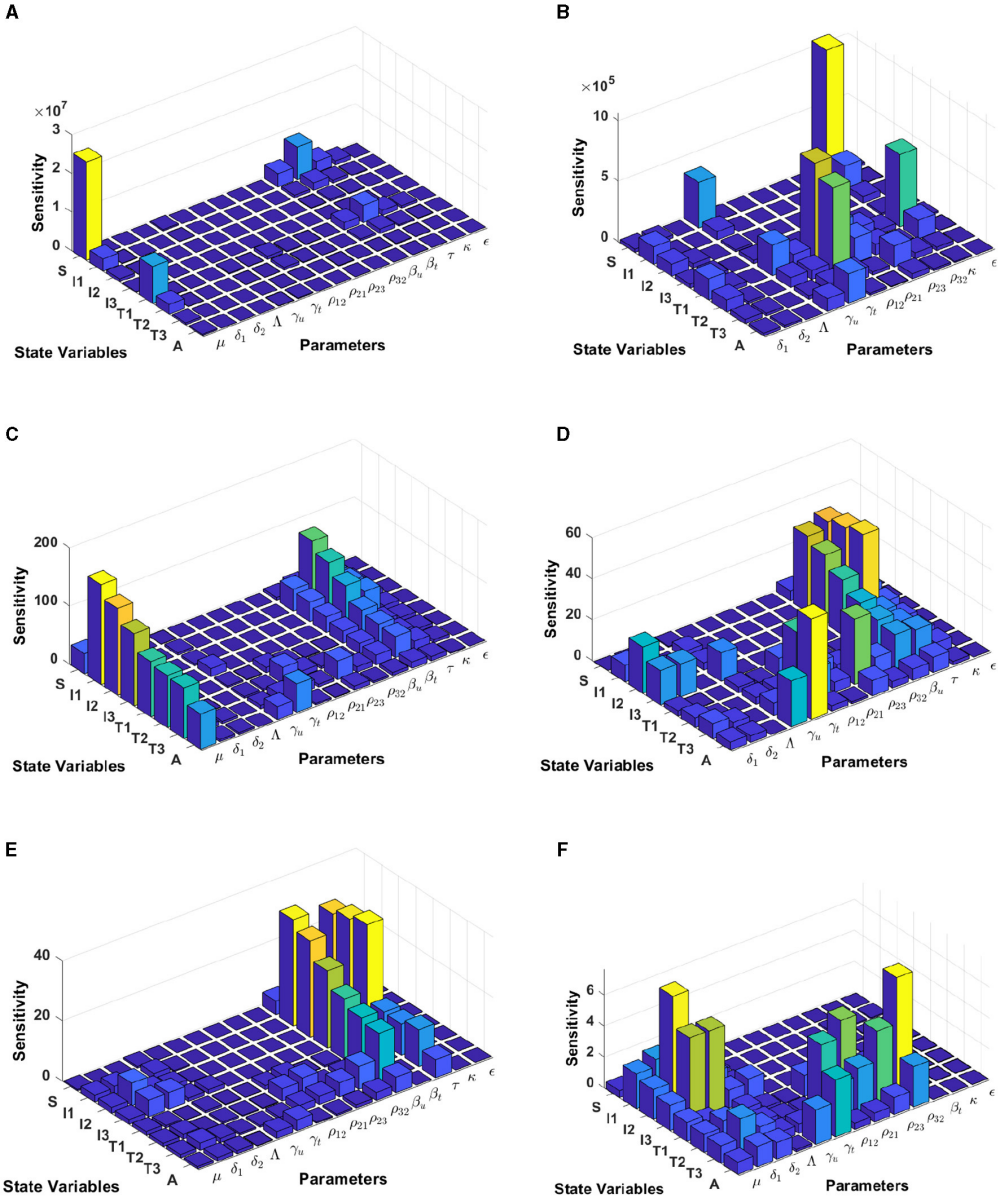
Local sensitivity analysis with non-normalization **(A, B)**, half-normalization **(C, D)**, and full-normalization **(E, F)** techniques using the best-fit parameter for Eswatini.

In this computational simulation, results from Figure 6 are computed by using incidence data from Eswatini with the model initial populations *S*(0) = 854, 011, *I*_1_(0) = 9, 529, *I*_2_(0) = 11, *I*_3_(0) = 7, 970, *T*_1_(0) = 63, *T*_2_(0) = 3, 214, *T*_3_(0) = 8, 920, and *A*(0) = 1, 404, and the estimated model parameters are *μ* = 0.013, δ_1_ = 0.33, δ_2_ = 0.34, Λ = 11861.26, *γ*_*u*_ = 0.1, *γ*_*t*_ = 0.018, *ρ*_12_ = 0.1462, *ρ*_21_ = 0.57, *ρ*_23_ = 0.1462, *ρ*_32_ = 0.82, *β*_*u*_ = 0.9529, *β*_*t*_ = 0.001, *τ* = 0.7970, *κ* = 0.0063, and *ϵ* = 0.3214

When we use the non-normalization technique, it can be seen that the model states *S* and *T*_1_ are very sensitive to *μ*, *β*_*t*_, *β*_*u*_, *κ*, and *ρ*_12_, while all model states are less sensitive to the model parameters δ_2_, Λ, *ρ*_32_, and *ϵ*; see panels (A, B). Furthermore, using the half-normalization method shows that almost all model states are very sensitive to the model parameters *μ*, *β*_*t*_, *β*_*u*_, and *τ*, whereas they are less sensitive to the other model parameters; see panels (C, D). Interestingly, applying the full-normalization method shows that almost all model variables are sensitive to *β*_*u*_ and *τ*, while the other variables have different sensitivities to the model parameters, this is clearly seen in panels (E, F).

#### 3.5.2 Model sensitivity analysis in Lesotho

**Figure 7:**
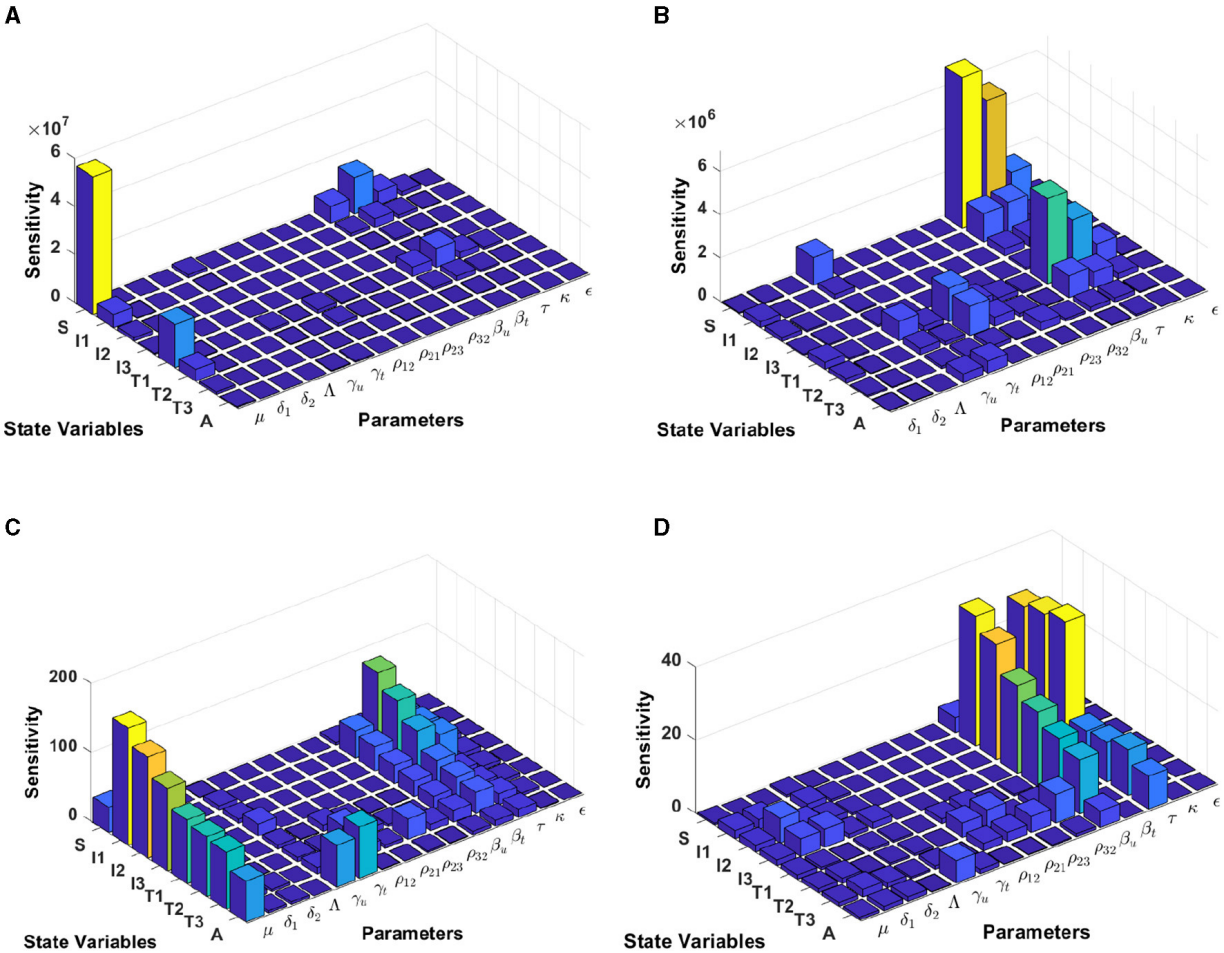
Local sensitivity analysis with non-normalization **(A, B)**, half-normalization **(C)**, and full-normalization **(D)** techniques using the best-fit parameter for Lesotho.

The results from Figure 7 are computed by using incidence data from Lesotho with the model initial populations *S*(0) = 1, 799, 000, *I*_1_(0) = 3, 676, *I*_2_(0) = 5, 018, *I*_3_(0) = 37, 272, *T*_1_(0) = 3, 420, *T*_2_(0) = 1, 701, *T*_3_(0) = 1, 982, and *A*(0) = 1, 163, and the estimated model parameters are *μ* = 0.013, δ_1_ = 0.33, δ_2_ = 0.34, Λ = 1, 799, 000/72, *γ*_*u*_ = 0.1, *γ*_*t*_ = 0.018, *ρ*_12_ = 0.1462, *ρ*_21_ = 0.57, *ρ*_23_ = 0.1462, *ρ*_32_ = 0.82, *β*_*u*_ = 0.8999, *β*_*t*_ = 0.0001, *τ* = 0.7909, *κ* = 0.0131, and *ϵ* = 0.2710

By using the non-normalization technique, it shows that the model states *S* and *T*_1_ are very sensitive to *μ*, *β*_*t*_, *β*_*u*_ and *τ*, while the other model states are less sensitive to the model parameters; see panels (A, B). When we use the half-normalization method, it shows that almost all model states are very sensitive to the model parameters *μ*, *β*_*t*_, *β*_*u*_, and *τ*, whereas they are less sensitive to the other model parameters; see panel (C). Furthermore, by applying the full-normalization method shows that almost all model variables are sensitive to *β*_*u*_ and *τ*, while we can also observe that there are different levels of sensitivities between model variables and parameters; see panel (D).

#### 3.5.3 Model sensitivity analysis in Botswana

**Figure 8:**
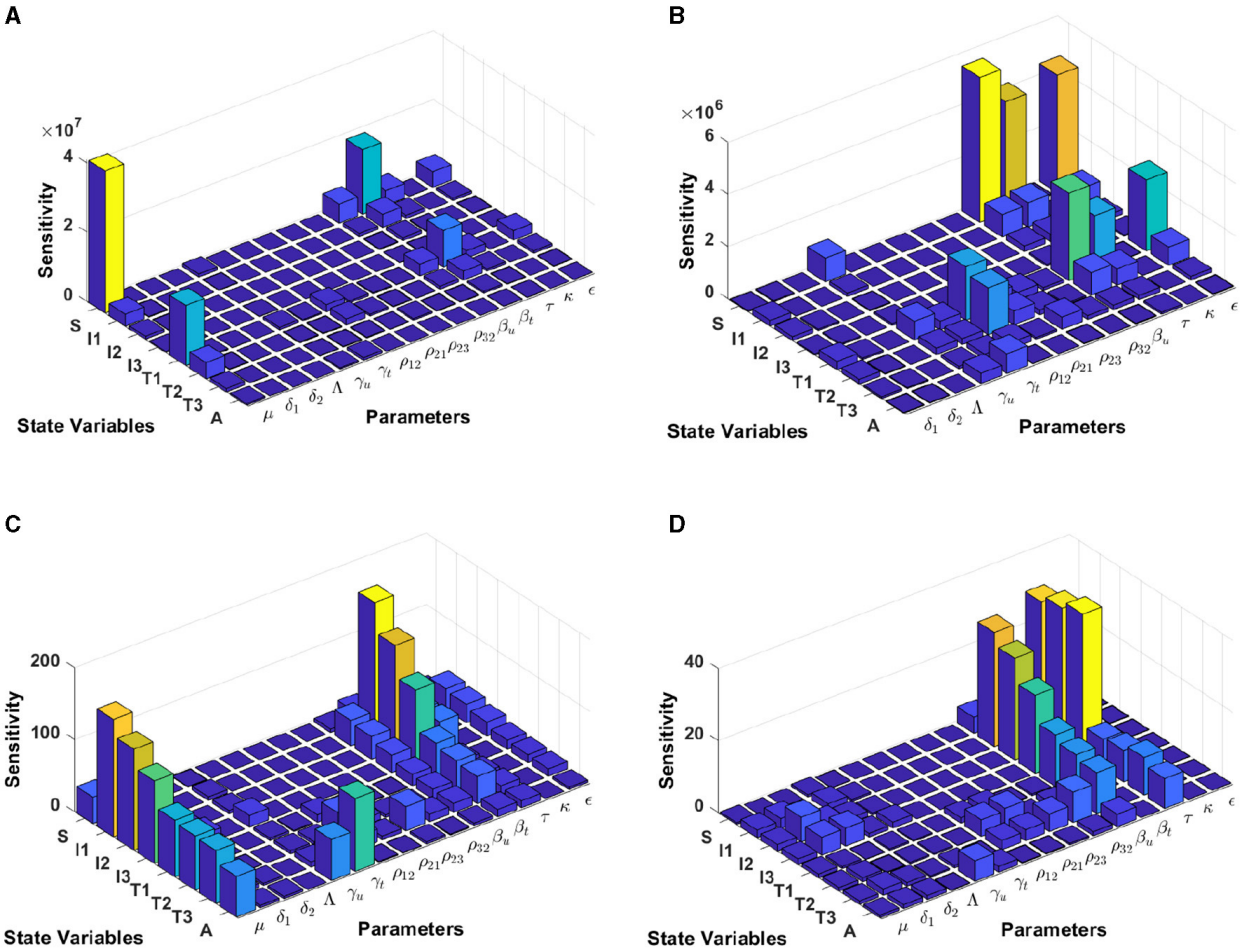
Local sensitivity analysis with non-normalization **(A, B)**, half-normalization **(C)**, and full-normalization **(D)** techniques using the best-fit parameter for Botswana.

Using the model initial states and estimated model parameters in computational simulations, results from Figure 8 are computed by using incidence data from Botswana with the model initial populations *S*(0) = 1, 341, 000, *I*_1_(0) = 17, 426, *I*_2_(0) = 3, 379, *I*_3_(0) = 39, 459, *T*_1_(0) = 31, 384, *T*_2_(0) = 7, *T*_3_(0) = 14, 015, and *A*(0) = 1, 243, and the estimated model parameters are *μ* = 0.013, δ_1_ = 0.33, δ_2_ = 0.34, Λ = 1341000/72, *γ*_*u*_ = 0.1, *γ*_*t*_ = 0.018, *ρ*_12_ = 0.1462, *ρ*_21_ = 0.57, *ρ*_23_ = 0.1462, *ρ*_32_ = 0.82, *β*_*u*_ = 0.8991, *β*_*t*_ = 0.0001, *τ* = 0.8689, *κ* = 0.8974, and *ϵ* = 0.0067.

The results of the non-normalization technique shows that the model states *S* and *T*_1_ are very sensitive to *μ*, *β*_*t*_, *β*_*u*_, *τ*, and *ϵ*, while there are different levels of sensitivities between other model states and parameters; see panels (A, B). Using the half-normalization method shows that almost all model states are very sensitive to the model parameters *μ* and *β*_*t*_, whereas there are sensitivities to the other model parameters; see panel (C). Furthermore, applying the full-normalization method shows that almost all model variables are sensitive to *β*_*u*_ and *τ*, while we can also see that there are different levels of sensitivities between model variables and parameters; see Figure panel (D).

#### 3.5.4 Model sensitivity analysis in South Africa

**Figure 9:**
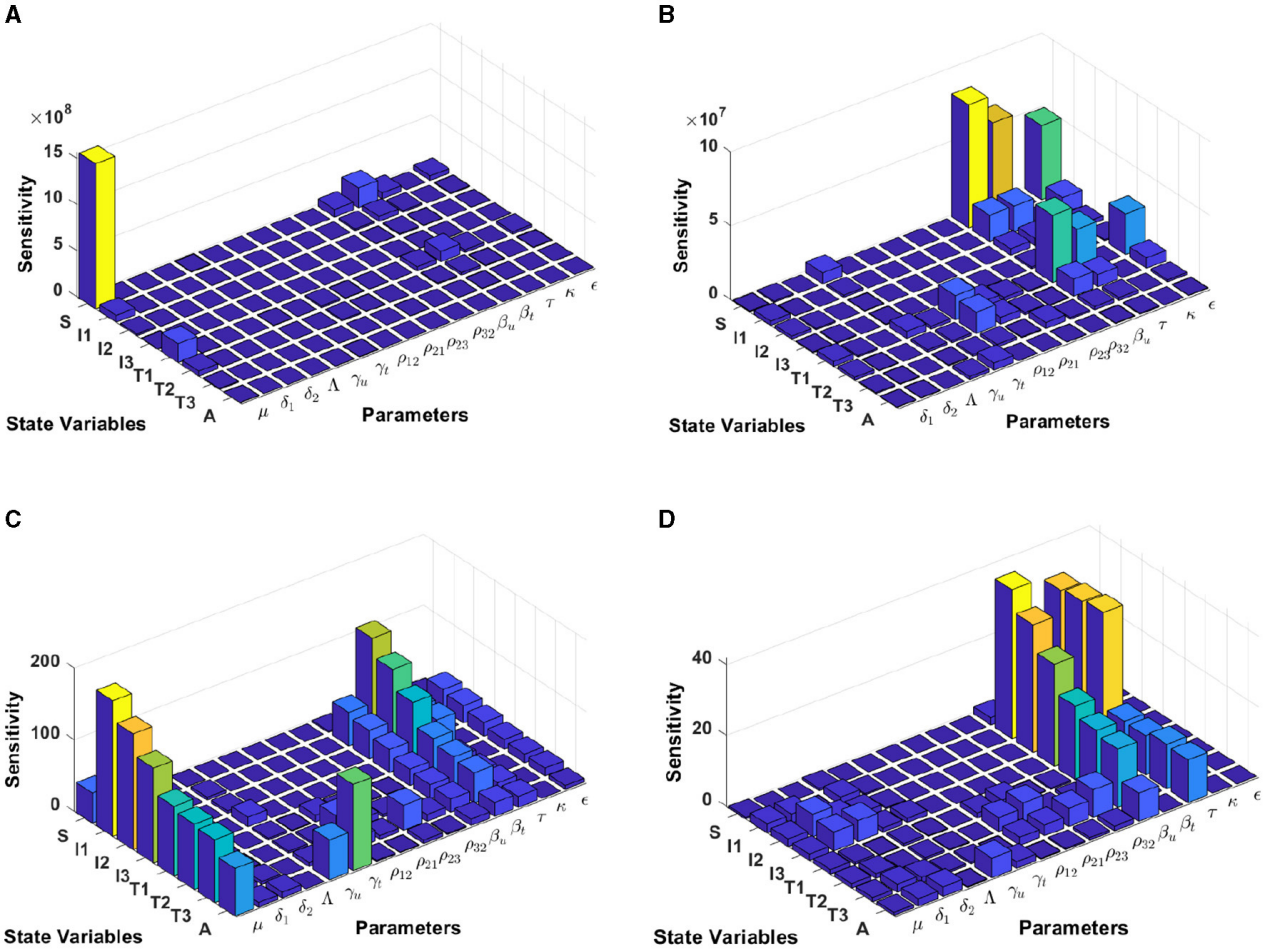
Local sensitivity analysis with non-normalization **(A, B)**, half-normalization **(C)**, and full-normalization **(D)** techniques using the best-fit parameter for South Africa.

Computational results shown in Figure 9 are computed by using incidence data from South Africa with the model initial populations *S*(0) = 39, 880, 000, *I*_1_(0) = 127, 900, *I*_2_(0) = 145, 386, *I*_3_(0) = 101, 214, *T*_1_(0) = 104, *T*_2_(0) = 197, 709, *T*_3_(0) = 99, 926, and *A*(0) = 5, 033, and the estimated model parameters are *μ* = 0.013, δ_1_ = 0.33, δ_2_ = 0.34, Λ = 39, 880, 000/72, *γ*_*u*_ = 0.1, *γ*_*t*_ = 0.018, *ρ*_12_ = 0.1462, *ρ*_21_ = 0.57, *ρ*_23_ = 0.1462, *ρ*_32_ = 0.82, *β*_*u*_ = 0.8999, *β*_*t*_ = 0.0001, *τ* = 0.7945, *κ* = 0.6706, and *ϵ* = 0.0001.

When we use the non-normalization technique, it can be seen that the model states *S* and *T*_1_ are very sensitive to *μ*, *β*_*t*_, *β*_*u*_.*κ*, and *ϵ*, while the other states are less sensitive to the other model parameters; see panels (A, B). Furthermore, using the half-normalization method shows that almost all model states are very sensitive to the model parameters *μ*, *β*_*t*_, *β*_*u*_, and *ϵ*, whereas they are less sensitive to the other model parameters; see panel (C). Interestingly, applying the full-normalization method shows that almost all model variables are sensitive to *β*_*u*_ and *τ*, while the other variables have different sensitivities to the model parameters, this is clearly seen in panel (D).

### 3.6 Autonomous simulation

In this simulation, we conduct the analysis using estimated parameters for Eswatini. We divide our simulation into three scenarios to understand the impact of the infection rate, the quality of prevention (condom use), and the effectiveness of massive detection. We employ bifurcation parameters and autonomous simulations at several sample points on the bifurcation diagram.

#### 3.6.1 Effect of infection rate *β*_*u*_

**Figure 10:**
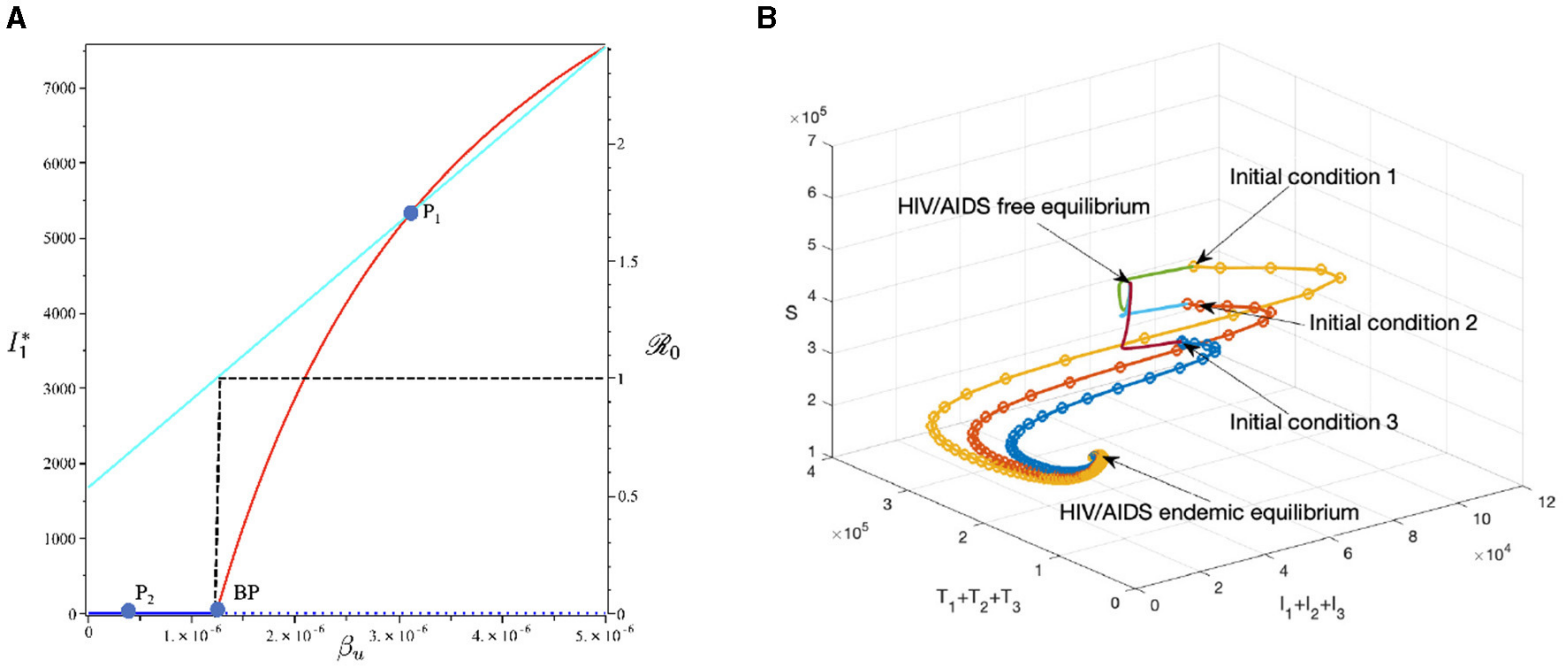
The bifurcation diagram of the system (3) with respect to *β*_*u*_ is presented in **(A)**. In this diagram, “BP" denotes the branch point occurring at *ℛ*_0_ = 1. The cyan, red, and blue curves represent *ℛ*_0_, the endemic equilibrium, and the HIV/AIDS-free equilibrium, respectively. Solid and dotted curves indicate stable and unstable equilibria, respectively. **(B)** Depicts the trajectories of total infected, total treated, and susceptible individuals tending to HIV/AIDS free-equilibrium point for *β*_*u*_ at *P*_1_ and to HIV/AIDS endemic equilibrium for *β*_*u*_ at *P*_2_.

To conduct the simulation, we set *β*_*u*_ as the bifurcation parameter, while the other parameters are the best-fit parameter for Eswatini (see Section 3.5.1). The numerical results are presented in Figure 10. It is clear to see that a larger value of *β*_*u*_ will increase *ℛ*_0_ and *I*_1_ in endemic equilibrium. Based on this dataset, we determine that *ℛ*_0_ = 1 when $\beta_{u}=1.23\times 10^{-6}$. At a sample point *P*_2_ ($\beta_{u}=0.4\times 10^{-6}$, *ℛ*_0_ = 0.687), we observe that the HIV/AIDS-free equilibrium point is stable, with the following values:


$$S=736,680,I_{1}=0,I_{2}=0,I_{3}=0,T_{1}=0,T_{2}=0,T_{3}=0,A=0.$$

On the other hand, at *P*_1_ ($\beta_{u}=3\times 10^{-6}$, we have *ℛ*_0_ = 1.66), we observe that the HIV/AIDS-endemic equilibrium is stable, with the following values:


$$\begin{array}{c} S=443342,I_{1}=5135,I_{2}=1361,I_{3}=142,T_{1}=15196,\\ T_{2}=23158,T_{3}=25493,\text{ and }A=16307. \end{array}$$

**Figure 11:**
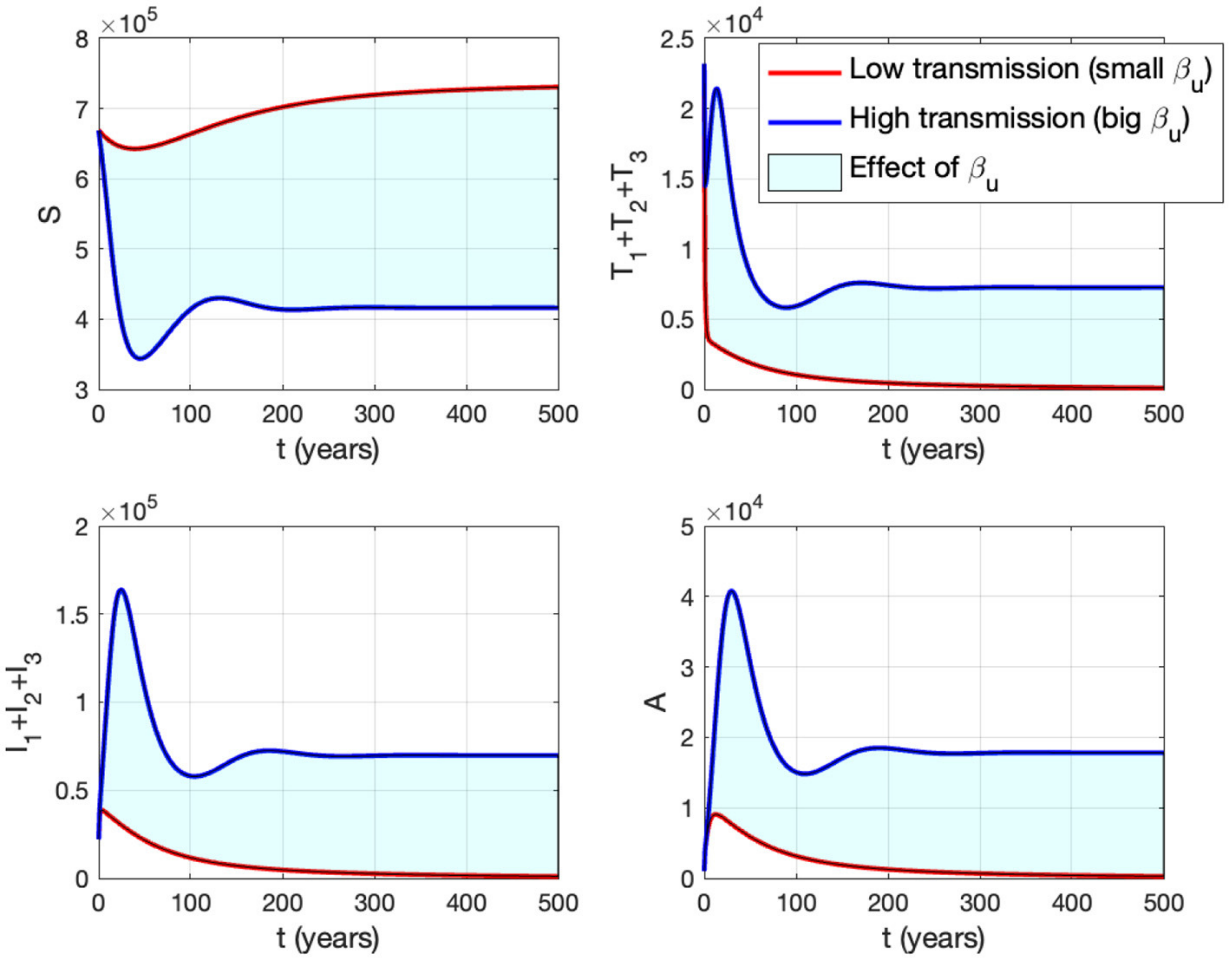
The impact of *β*_*u*_ on the dynamics of susceptible (up left), total infected (up right), total treated (below left), and AIDS (below right). The blue curve represents the dynamic for a low transmission rate *β*_*u*_ (1.488 × 10^−7^), while the red curve represents the dynamic for a high transmission rate *β*_*u*_ (1.488 × 10^−6^).

In panel (A) of Figure 10, it is evident that for $\beta_{u}<1.23\times 10^{-6}$, *ℛ*_0_ < 1, indicating the stability of the HIV/AIDS-free equilibrium. As *β*_*u*_ increases, *ℛ*_0_ also increases (as seen in the cyan curve). Upon reaching the branch point (BP), the HIV/AIDS-free equilibrium becomes unstable, leading to the stable HIV/AIDS-endemic equilibrium, which grows in significance as *β*_*u*_ continues to increase. Panel (B) illustrates how trajectories from different initial conditions converge toward the same stable equilibrium point, namely, the HIV/AIDS-free equilibrium or the HIV/AIDS-endemic equilibrium. Figure 11 shows the dynamic of the system (3) with respect to various values of *β*_*u*_. It can be seen that larger *β*_*u*_ will increase the number of infected individuals *I*_*i*_, *T*_*i*_, and *A*.

#### 3.6.2 The effect of proportion of the condom use (*κ*)

**Figure 12:**
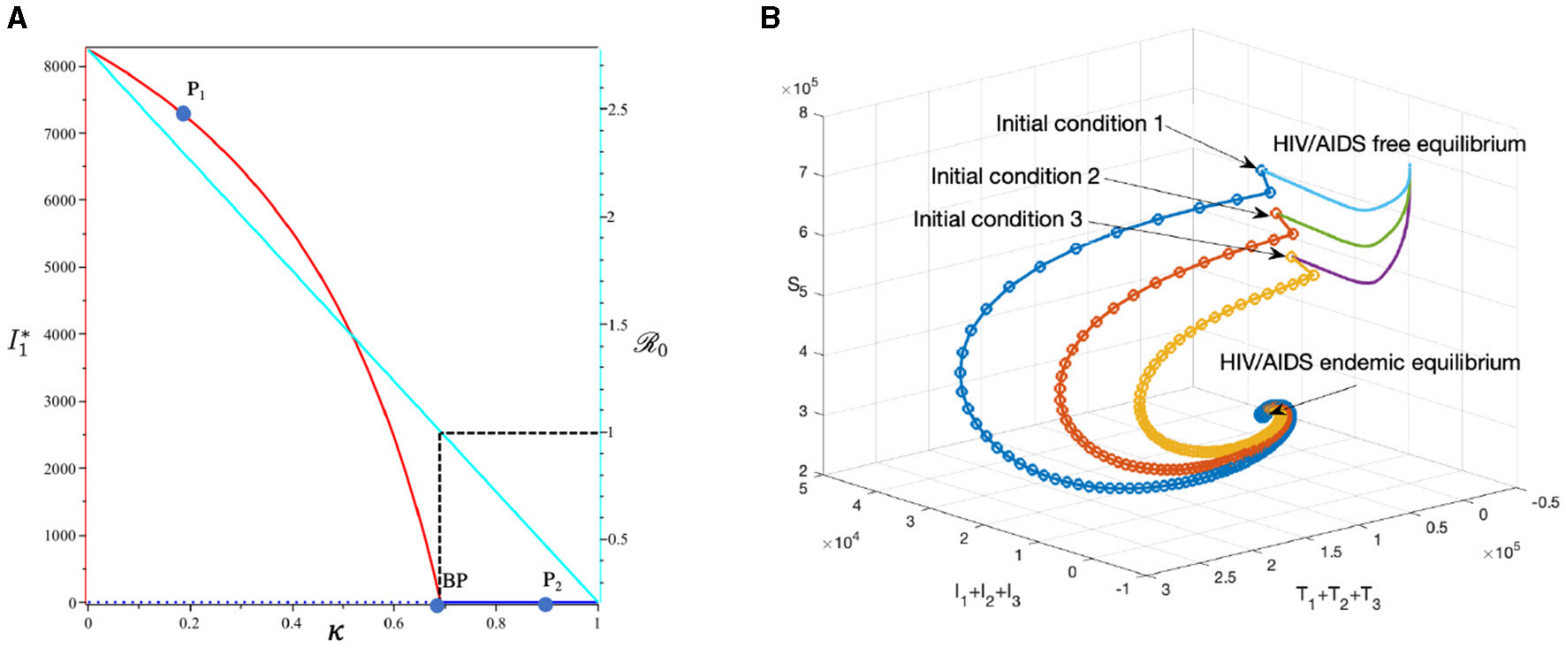
The bifurcation diagram of the system (3) with respect to *κ* is presented in **(A)**. In this diagram, “BP” denotes the branch point occurring at *ℛ*_0_ = 1. The cyan, red, and blue curves represent *ℛ*_0_, the endemic equilibrium, and the HIV/AIDS-free equilibrium, respectively. Solid and dotted curves indicate stable and unstable equilibria, respectively. **(B)** Depicts the trajectories of total infected, total treated, and susceptible individuals tending to HIV/AIDS-endemic equilibrium point for *κ* at *P*_1_ and to HIV/AIDS-free equilibrium for *κ* at *P*_2_.

This section is dedicated to examining the impact of the proportion of people who use condoms during sexual contact (*κ*) on reducing the HIV/AIDS infection rate. For this simulation, we use the same parameter values as in Section 3.6.1, with the exception of *κ*, which is varying. The numerical results are presented in Figure 12. With this data set, we determine that $R_{0}=1$ at *ϵ* = 0.691. As indicated by the cyan curve in panel (A), a higher quality of condoms (larger *ϵ*) leads to a smaller *ℛ*_0_. At sample point *P*_1_, with *ϵ* = 0.2, we have *ℛ*_0_ = 2.262, resulting in a stable HIV/AIDS-endemic equilibrium at


$$\begin{array}{c} S=325,640,I_{1}=7,195,I_{2}=1,908,I_{3}=199,T_{1}=21,293,\\ T_{2}=32,450,T_{3}=35,722,\text{ and }A=22851. \end{array}$$

On the other hand, at sample point *P*_2_, when *ϵ* = 0.9, we have *ℛ*_0_ = 0.464 which gives us a stable HIV/AIDS-free equilibrium point at


$$\begin{array}{c} S=736,680,I_{1}=0,I_{2}=0,I_{3}=0,T_{1}=0,T_{2}=0,\\ T_{3}=0,\text{ and }A=0. \end{array}$$

**Figure 13:**
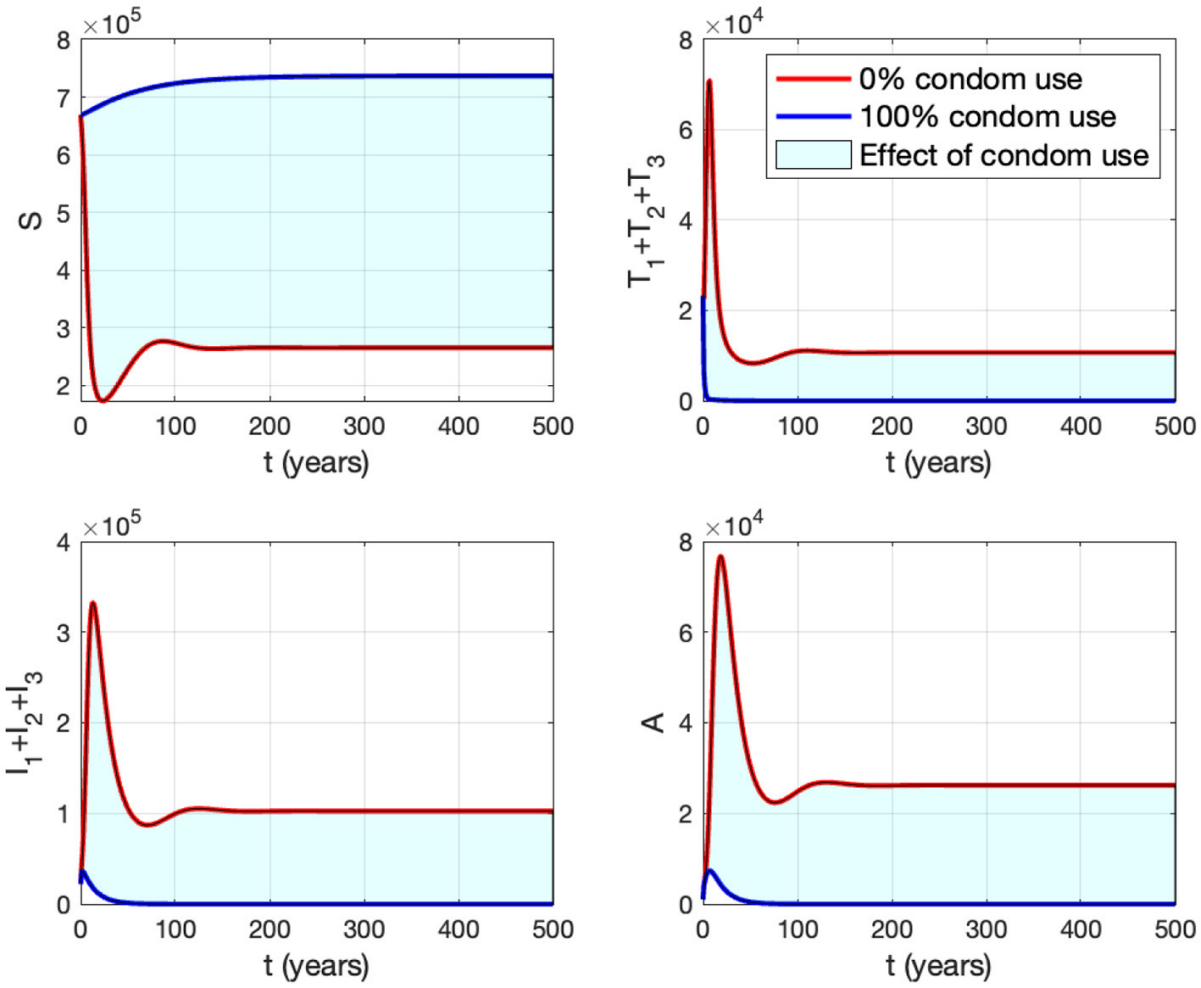
The impact of *κ* to the dynamics of susceptible (up left), total infected (up right), total treated (below left), and AIDS (below right). The red curve represents the dynamic when no individuals use condom, while the blue curve represents the dynamic when all people use condoms during sexual contact.

Panel (B) shows the trajectories of all solutions from different initial conditions tending toward their stable endemic equilibrium and disease-free equilibrium. Figure 13 shows the dynamic of the system (3) with respect to various values of *κ*. It can be seen that larger value of *κ* will reduce the number of infected individuals *I*_*i*_, *T*_*i*_, and *A*.

#### 3.6.3 The effect of massive case detection from *I*_1_ to *T*_1_ (*τ*_1_)

**Figure 14:**
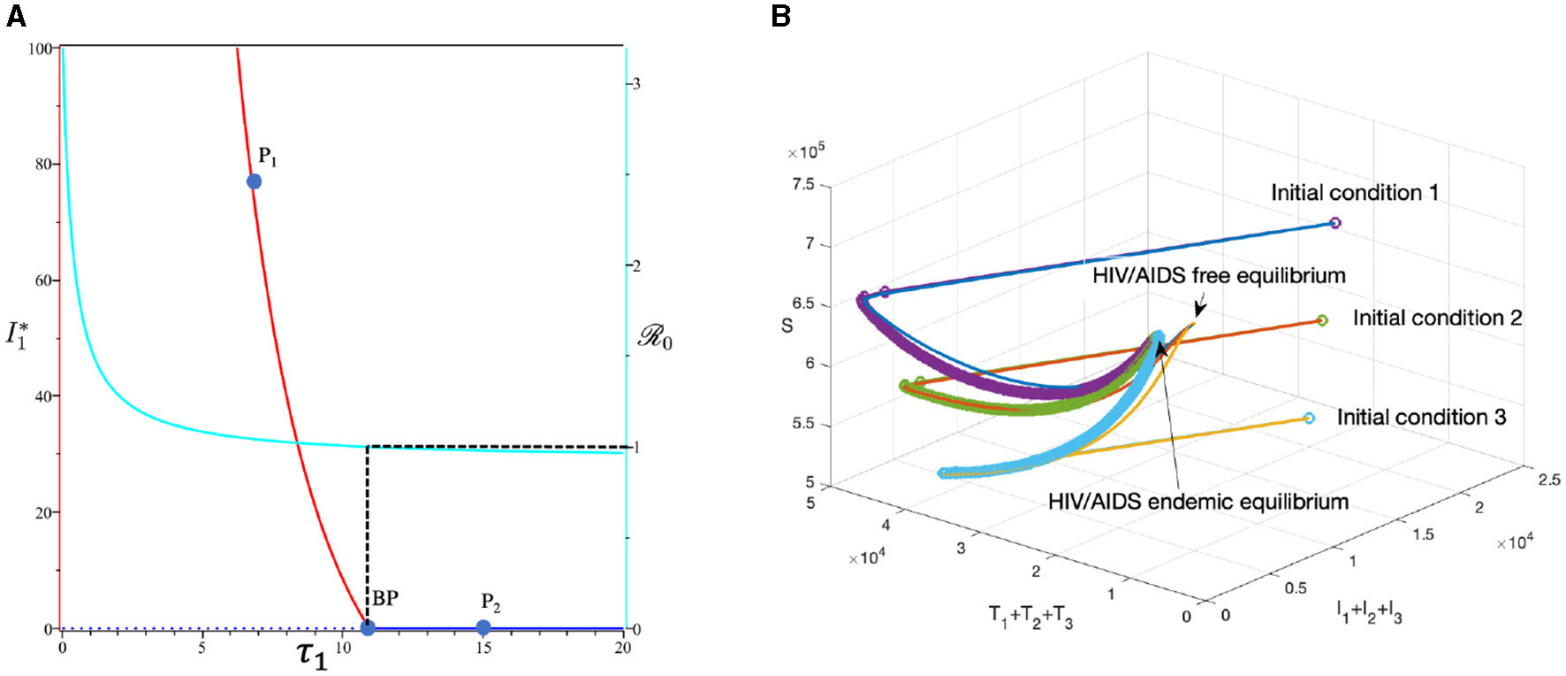
The bifurcation diagram of the system (3) with respect to *τ*_1_ is presented in **(A)**. In this diagram, “BP” denotes the branch point occurring at *ℛ*_0_ = 1. The cyan, red, and blue curves represent *ℛ*_0_, the endemic equilibrium, and the HIV/AIDS-free equilibrium, respectively. Solid and dotted curves indicate stable and unstable equilibria, respectively. **(B)** Depicts the trajectories of total infected, total treated, and susceptible individuals tending to HIV/AIDS-endemic equilibrium point for *τ*_1_ at *P*_1_ and to HIV/AIDS-free equilibrium for *τ* at *P*_2_.

This section is dedicated to exploring the impact of the rate of case detection (*τ*_1_) on the acceleration of treatment for individuals. In this simulation, we employ data for Eswatini, with the exception of *τ*_1_, which serves as a freely adjustable bifurcation parameter. The numerical findings are presented in Figure 14. Within this dataset, we reveal the refined result that $R_{0}=1$ when *τ*_1_ = 10.93. As described by the cyan curve in panel (A), a more intensive case detection (characterized by a larger *τ*_1_) corresponds to a smaller *ℛ*_0_. At sample point *P*_1_, *τ* = 7 gives *ℛ*_0_ = 1.04 and gives us the following endemic equilibrium.


$$\begin{array}{c} =708,205,I_{1}=68,I_{2}=18,I_{3}=2,T_{1}=1,662,T_{2}=2,343,\\ T_{3}=2,546,\text{ and }A=1,597. \end{array}$$

On the other hand, at sample point *P*_2_, when *τ* = 15, we have *ℛ*_0_ = 0.98, which gives us a stable HIV/AIDS-free equilibrium point at


$$\begin{array}{c} S=736,680,I_{1}=0,I_{2}=0,I_{3}=0,T_{1}=0,\\ T_{2}=0,T_{3}=0,\text{ and }A=0. \end{array}$$

**Figure 15:**
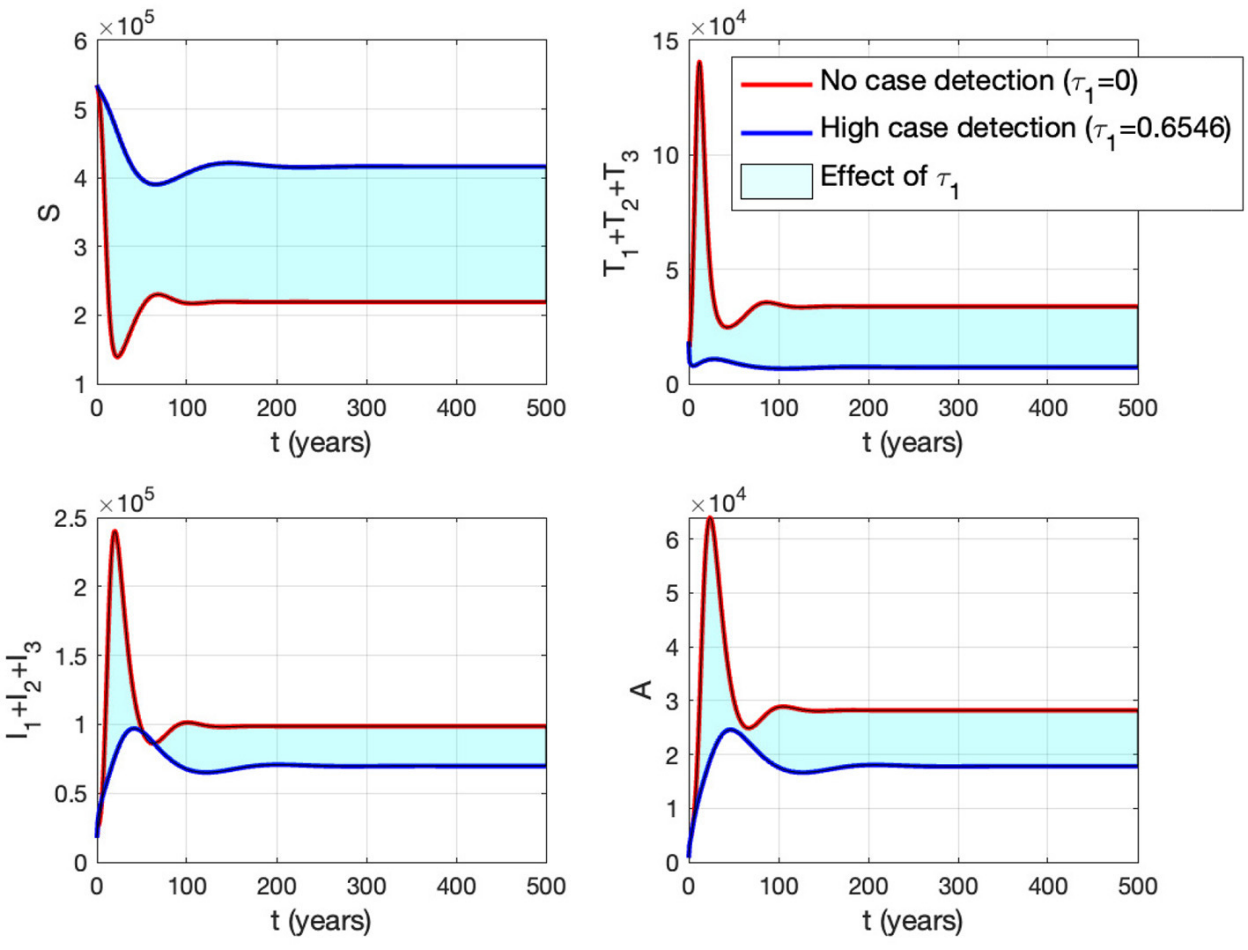
The impact of *τ*_1_ on the dynamics of susceptible (up left), total infected (up right), total treated (below left), and AIDS (below right). The red curve represents when no early case detection was implemented, while the blue curve represents when it was implemented.

Panel (B) shows the trajectory of all solutions from different initial conditions tends toward either how stable endemic equilibrium or disease-free equilibrium. Figure 15 shows the dynamic of the system (3) with respect to various values of *τ*_1_. It can be seen that a larger value of *τ* will reduce the number of infected individuals *I*_*i*_, *T*_*i*_, and *A*.

### 3.7 Minimum proportion on the use of condoms to eliminate HIV/AIDS

**Figure 16:**
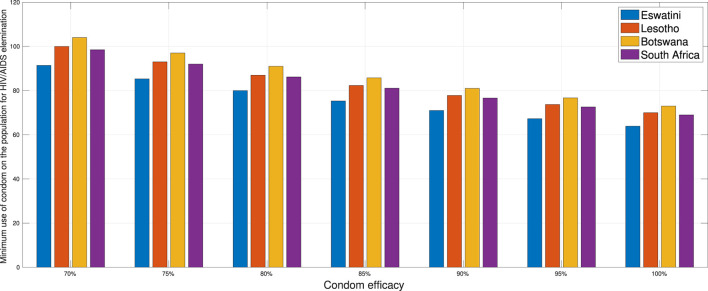
A comparison of the minimum population percentage in each country required for condom usage, contingent upon the quality of the condom.

In this section, we conduct numerical experiments to understand how the proportion of people using condoms impacts the spread of HIV/AIDS. We use four different datasets representing four countries: Eswatini, Lesotho, Botswana, and South Africa. All parameters are consistent with those in Appendix 2, except for condom quality (*ϵ*). We calculate the minimum population proportion that needs to use condoms with a specific condom quality (between 70 and 100%) to achieve *ℛ*_0_ < 1. The results are depicted in Figure 16. It is clear that better condom quality requires a smaller proportion of the population to reduce *ℛ*_0_ to less than one. Based on the analysis results, an efficacy of 70% for condoms indicates that Eswatini could eliminate HIV/AIDS if a minimum of 91.39% of the infected population consistently uses condoms during sexual activities. Simultaneously, with the same efficacy, it is evident that Lesotho would need to mandate 100% condom usage to achieve the same goal, while Botswana cannot solely rely on condom use to eliminate HIV/AIDS. If condom efficacy improves, for instance, reaching 90%, the proportion of the infected population required to use condoms during sexual activities decreases. It would be 71% for Eswatini and up to 81% for Botswana.

## 4 Extension of the HIV/AIDS model as an optimal control problem

### 4.1 Optimal control model

In this section, we expand our model from the system (3) to an optimal control problem by introducing time-dependent variables *u*_1_(*t*) to represent the proportion of condom use (*κ*) and the case detection rate (*τ*_1_, *τ*_2_, *τ*_3_), which is now denoted by *u*_2_(*t*), *u*_3_(*t*), *u*_4_(*t*), respectively. Consequently, our model now reads as follows:


(4) $$\begin{array}{ll} \; & \frac{dS}{dt}=\Lambda -((1-\epsilon u_{1}(t))[\beta_{u}(I_{1}+I_{2}+I_{3})+\\ \; & \beta_{t}(T_{1}+T_{2}+T_{3})]+\mu )S,\\ \frac{dI_{1}}{dt} & =(1-\epsilon u_{1}(t))[\beta_{u}(I_{1}+I_{2}+I_{3})+\\ \; & \beta_{t}(T_{1}+T_{2}+T_{3})]S-(u_{2}(t)+\delta_{1}+\mu )I_{1},\\ \frac{dI_{2}}{dt} & =\delta_{1}I_{1}-(u_{3}(t)+\delta_{2}+\mu )I_{2},\\ \frac{dI_{3}}{dt} & =\delta_{2}I_{2}-(u_{4}(t)+\gamma_{u}+\mu )I_{3},\\ \frac{dT_{1}}{dt} & =u_{2}(t)I_{1}+(1-q)\rho T_{2}-(s\rho +\mu )T_{1},\\ \frac{dT_{2}}{dt} & =u_{3}(t)I_{2}+s\rho T_{1}+(1-r)\rho T_{3}-((1-q)\rho +q\rho +\mu )T_{2},\\ \frac{dT_{3}}{dt} & =u_{4}(t)I_{3}+q\rho T_{2}-((1-r)\rho +r\gamma_{t}+\mu )T_{3},\;\text{and}\\ \frac{dA}{dt} & =r\gamma_{t}T_{3}+\gamma_{u}I_{3}-(\mu +\eta )A, \end{array}$$

The objective of this optimal control approach is to minimize the number of untreated infected individuals *I*_1_, *I*_2_, and *I*_3_, as well as *A*, by optimizing the intervention of the proportion of condom use (*u*_1_) and the case detection rate (*u*_2_, *u*_3_, and *u*_4_). Therefore, the cost function reads as follows:


(5) $$\begin{array}{l} J(u_{1},u_{2})=\int_{0}^{t_{f}}(\omega_{1}u_{1}^{2}+\omega_{2}u_{2}^{2}+\omega_{3}u_{3}^{2}+\omega_{4}u_{4}^{4}+\\ \varphi_{1}I_{1}+\varphi_{2}I_{2}+\varphi_{3}I_{3}+\varphi_{4}A)dt, \end{array}$$

where *ω*_*i*_ for *i* = 1, 2, 3, and 4 and φ_*j*_ for *j* = 1, 2, 3, and 4 are the positive weight parameter for each component on J.

### 4.2 Characterization of the problem

We define the Hamiltonian of our problem as follows:


$$\begin{array}{l} ℋ=\omega_{1}u_{1}^{2}+\omega_{2}u_{2}^{2}+\omega_{3}u_{3}^{2}+\omega_{4}u_{4}^{2}+\varphi_{1}I_{1}+\varphi_{2}I_{2}+\varphi_{3}I_{3}+\varphi_{4}A\\ \;\;\;\;+\lambda_{1}(\Lambda -((1-\epsilon u_{1}(t))[\beta_{u}(I_{1}+I_{2}+I_{3})+\beta_{t}(T_{1}+T_{2}+T_{3})]\\ \;\;\;\;+\mu )S)\\ \;\;\;\;+\lambda_{2}((1-\epsilon u_{1}(t))[\beta_{u}(I_{1}+I_{2}+I_{3})+\beta_{t}(T_{1}+T_{2}+T_{3})]S\\ \;\;\;\;-(u_{2}(t)+\delta_{1}+\mu )I_{1})\\ \;\;\;\;+\lambda_{3}(\delta_{1}I_{1}-(u_{3}(t)+\delta_{2}+\mu )I_{2})+\lambda_{4}(\delta_{2}I_{2}-(u_{4}(t)+\gamma_{u}+\mu )\\ \;\;\;\;I_{3})\\ \;\;\;+\lambda_{5}(u_{2}(t)I_{1}+(1-q)\rho T_{2}-(s\rho +\mu )T_{1})\\ \;\;\;+\lambda_{6}(u_{3}(t)I_{2}+s\rho T_{1}+(1-r)\rho T_{3}-((1-q)\rho +q\rho +\mu )T_{2})\\ \;\;\;+\lambda_{7}(u_{4}(t)I_{3}+q\rho T_{2}-((1-r)\rho +r\gamma_{t}+\mu )T_{3})\\ \;\;\;+\lambda_{8}(r\gamma_{t}T_{3}+\gamma_{u}I_{3}-(\mu +\eta )A). \end{array}$$

First, by taking the partial derivative of H with respect to each variable, the adjoint system of our problem is given as follows:


(6) $$\begin{array}{l} \frac{d\lambda_{1}}{dt}=-\frac{\partial H}{\partial S}\\ \;=\left(1-\epsilon u_{1}\right)\left(\beta_{u}\left(I_{1}+I_{2}+I_{3}\right)+\beta_{t}\left(T_{1}+T_{2}+T_{3}\right)\right)\\ \;\left(\lambda_{1}-\lambda_{2}\right)+\mu \lambda_{1},\\ \frac{d\lambda_{2}}{dt}=-\frac{\partial H}{\partial I_{1}}\\ \;=-\varphi_{1}+\left(1-\epsilon u_{1}\right)\beta_{u}S\left(\lambda_{1}-\lambda_{2}\right)+\delta_{1}\left(\lambda_{2}-\lambda_{3}\right)+\\ \;u_{2}\left(\lambda_{2}-\lambda_{5}\right)+\mu \lambda_{2},\\ \frac{d\lambda_{3}}{dt}=-\frac{\partial H}{\partial I_{2}}\\ \;=-\varphi_{2}+\left(1-\epsilon u_{1}\right)\beta_{u}S\left(\lambda_{1}-\lambda_{2}\right)+u_{3}\left(\lambda_{3}-\lambda_{6}\right)+\\ \;\delta_{2}\left(\lambda_{3}-\lambda_{4}\right)+\mu \lambda_{3},\\ \frac{d\lambda_{4}}{dt}=-\frac{\partial H}{\partial I_{3}}\\ \;=-\varphi_{3}+\left(1-\epsilon u_{1}\right)\beta_{u}S\left(\lambda_{1}-\lambda_{2}\right)+u_{4}\left(\lambda_{4}-\lambda_{7}\right)+\\ \;\gamma_{u}\left(\lambda_{4}-\lambda_{8}\right)+\mu \lambda_{4},\\ \frac{d\lambda_{5}}{dt}=-\frac{\partial H}{\partial T_{1}}\\ \;=\left(1-\epsilon u_{1}\right)\beta_{t}S\left(\lambda_{1}-\lambda_{2}\right)+s\rho \left(\lambda_{5}-\lambda_{6}\right)+\mu \lambda_{5},\\ \frac{d\lambda_{6}}{dt}=-\frac{\partial H}{\partial T_{2}}\\ \;=\left(1-\epsilon u_{1}\right)\beta_{t}S\left(\lambda_{1}-\lambda_{2}\right)+\left(1-q\right)\rho \left(\lambda_{6}-\lambda_{5}\right)+\\ \;q\rho \left(\lambda_{6}-\lambda_{7}\right)+\mu \lambda_{6},\\ \frac{d\lambda_{7}}{dt}=-\frac{\partial H}{\partial T_{3}}\\ \;=\left(1-\epsilon u_{1}\right)\beta_{t}S\left(\lambda_{1}-\lambda_{2}\right)+\left(1-r\right)\rho \left(\lambda_{7}-\lambda_{6}\right)+\\ \;r\gamma_{t}\left(\lambda_{7}-\lambda_{8}\right)+\mu \lambda_{7},\\ \frac{d\lambda_{8}}{dt}=-\frac{\partial H}{\partial A}\\ \;=-\varphi_{4}+\left(\mu +\eta \right)\lambda_{8}, \end{array}$$

completed with the transversality condition λ_*i*_(*t*_*f*_) = 0 for *i* = 1, 2, …8. The optimality condition is taken from $\frac{\partial H}{\partial u_{i}}=0$ for *i* = 1, 2, 3, and 4. Hence, taking this into account with the lower and upper bounds for *u*_*i*_, we have the optimal control variables that should satisfy as follows:


(7a) $$\begin{array}{l} u_{1}^{*}=\min\left\{\max\left\{u_{1}^{\min},\frac{\epsilon S\left(\lambda_{2}-\lambda_{1}\right)\left(\beta_{u}\left(I_{1}+I_{2}+I_{3}\right)+\beta_{t}\left(T_{1}+T_{2}+T_{3}\right)\right)}{2\omega_{1}}\right\},u_{1}^{\max}\right\}, \end{array}$$

(7b) $$\begin{array}{l} u_{2}^{*}=\min\left\{\max\left\{u_{2}^{\min},\frac{I_{1}\left(\lambda_{2}-\lambda_{5}\right)}{2\omega_{2}}\right\},u_{2}^{\max}\right\}, \end{array}$$

(7c) $$\begin{array}{l} u_{3}^{*}=\min\left\{\max\left\{u_{3}^{\min},\frac{I_{2}\left(\lambda_{3}-\lambda_{6}\right)}{2\omega_{3}}\right\},u_{3}^{\max}\right\},\text{ and} \end{array}$$

(7d) $$\begin{array}{ll} u_{4}^{*} & =\min\left\{\max\left\{u_{4}^{\min},\frac{I_{3}\left(\lambda_{4}-\lambda_{7}\right)}{2\omega_{4}}\right\},u_{4}^{\max}\right\}. \end{array}$$

To summarize our problem, we want to minimize the cost function given in Equation (5) subject to the state system in Equation (4) completed with its initial condition, the adjoint system in Equation (6) completed with its transversality condition, and the optimality condition in Equation (7). We use a forward-backward iterative method to solve the problem. We begin by giving an intial guess for the control variables for all *t* and using it to solve the state system in Equation (4) forward in time. Then, we solve the adjoint system in Equation (6) backward in time with the given transversality condition. Hence, with these results, we can update the optimal control value using the formula in Equation (7). We goback to the first step until the convergence criteria are achieved, which in our case is $|J^{\text{iteration-(i + 1)}}-J^{\text{iteration-(i)}}|<10^{-5}$.

### 4.3 Numerical experiments

The simulation in this section was conducted using parameter values corresponding to the best-fit values for Eswatini. Please refer to Appendix 2 for details. Furthermore, we have set the value for the weight parameter on the cost function as follows:


$$\omega_{1}=10^{5},\omega_{2}=\omega_{3}=\omega_{4}=5\times 10^{5},\varphi_{1}=\varphi_{2}=\varphi_{3}=\varphi_{4}=10,$$

and the initial condition given by


$$\begin{array}{l} \left[S\left(0\right),I_{1}\left(0\right),I_{2}\left(0\right),I_{3}\left(0\right),T_{1}\left(0\right),T_{2}\left(0\right),T_{3}\left(0\right),A\left(0\right)\right]=\\ \left[3.62,0.1,0.02,0.002,0.358,0.559,0.625,0.4\right]\times 10^{5}. \end{array}$$

In the following, we set three scenarios for the implementation of controls.

- **Scenario 1**: The implementation of condom use (*u*_1_) only, while case detection (*u*_2_) set to be zero. Hence *u*_1_ ≠ 0 and *u*_2_ = *u*_3_ = *u*_4_ = 0.
- **Scenario 2**: The implementation of case detection (*u*_2_, *u*_3_, *u*_4_) only, while condom use (*u*_1_) set to be zero. Hence *u*_1_ = 0, *u*_2_ ≠ 0, *u*_3_ ≠ 0, and *u*_4_ ≠ 0.
- **Scenario 3**: The combination of condom use (*u*_1_) and case detection (*u*_2_, *u*_3_, and *u*_4_). Hence *u*_1_ ≠ 0, *u*_2_ ≠ 0, *u*_3_ ≠ 0, and *u*_4_ ≠ 0.

#### Scenario 1

**Figure 17:**
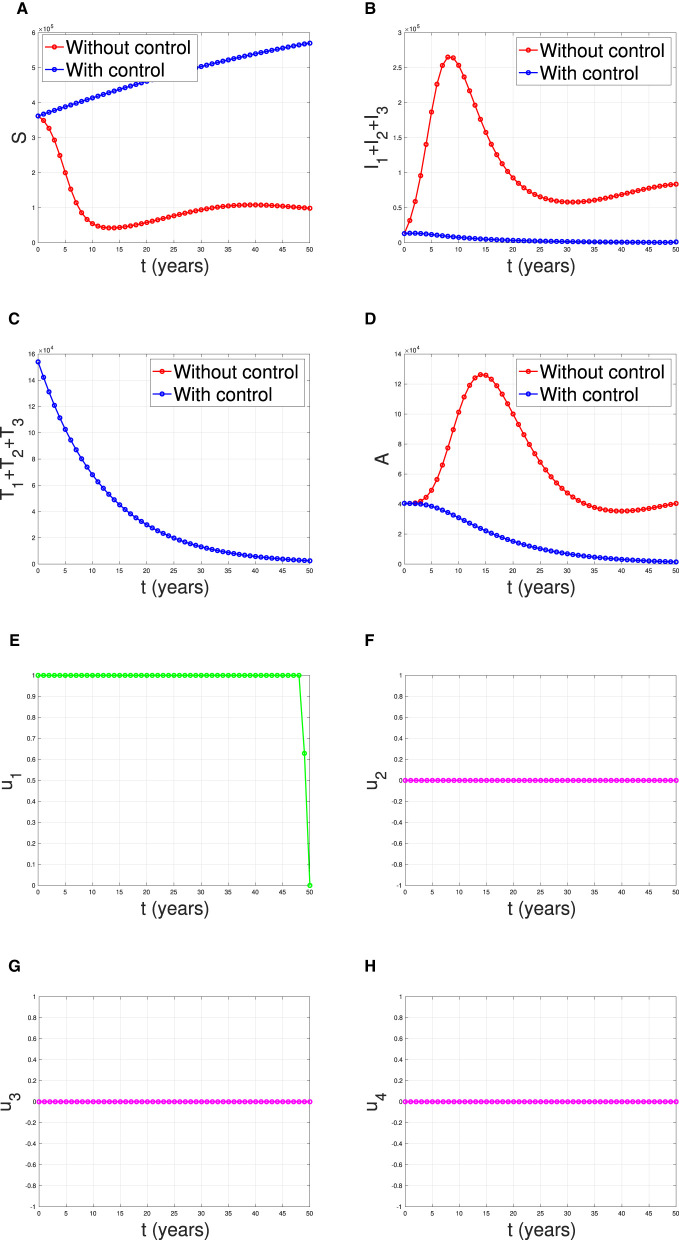
The dynamic of model output for **(A)** susceptible, **(B)** total PLHIV untreated *I*, **(C)** total PLHIV receiving treatment *T*, **(D)** total PLHIV with AIDS ilness *A*, and **(E–H)** for control variables under the scenario when condom use used as a single intervention.

The first numerical experiment involves the use of condoms as the sole strategy to prevent the spread of HIV/AIDS. The results are presented in Figure 17 where the dynamics of model output are given in panels (A–D) while the dynamics of control are shown in panels (E–H). It is clear to see that intervention in condom usage should be given almost maximal effort from the beginning of the simulation. Since condom usage can prevent new infections, we can observe an increase in the number of susceptible individuals [panel (A)] and significant decreases in the total number of PLHIV without treatment (total of *I*_*i*_) and PLHIV with AIDS illness (*A*). Since the intervention of case detection was not used, we can see that the number of undetected case decreases and tends to zero [see panel (C)]. The total number of infections averted using this strategy is 7.59 × 10^6^ at an optimal cost of 2.06 × 10^11^.

#### Scenario 2

**Figure 18:**
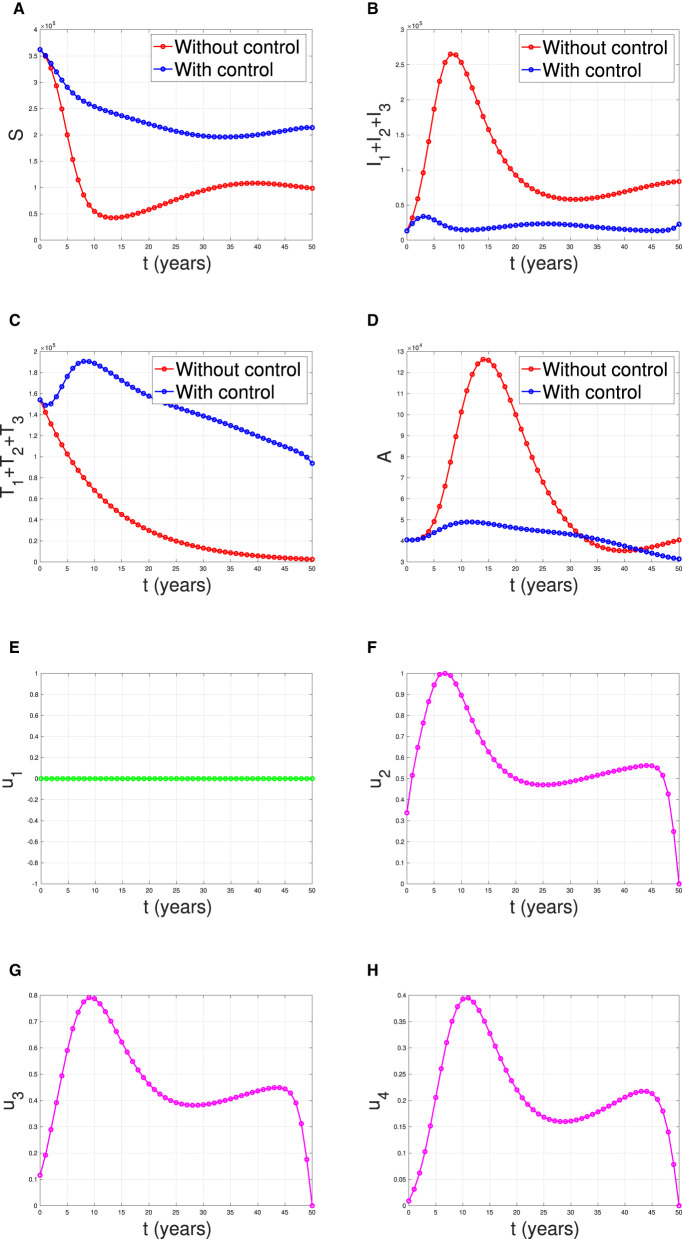
The dynamic of model output for **(A)** susceptible, **(B)** total PLHIV untreated *I*, **(C)** total PLHIV receiving treatment *T*, **(D)** total PLHIV with AIDS illness *A*, and **(E–H)** for control variables under the scenario when case detection used as a single intervention.

Figure 18 illustrates the situation when the government relied solely on the implementation of case detection as a single intervention (*u*_2_, *u*_3_, *u*_4_ only). The dynamics of control are depicted in panels (F–H). The intervention is applied with high intensity at the beginning of the simulation and then decreasing to an almost constant for a short period and decreases again when the final time approaches. With this strategy in place, we can observe a decrease in the number of PLHIV without and with treatment [panels (B, C)], resulting in a reduced number of individuals with AIDS [panel (D)]. The total number of infected individuals averted using this strategy is 3.74 × 10^4^ at an optimal cost of 2.52 × 10^16^.

#### Scenario 3

**Figure 19:**
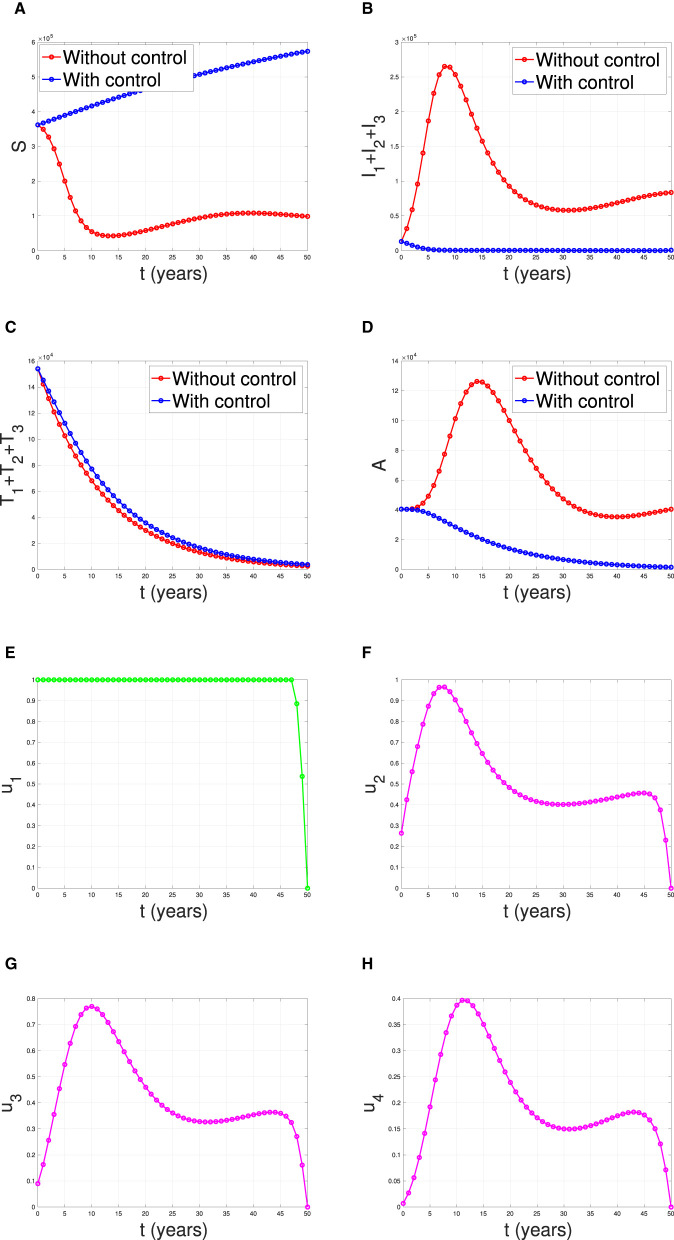
The dynamic of model output for **(A)** Susceptible, **(B)** total PLHIV untreated *I*, **(C)** total PLHIV receiving treatment *T*, **(D)** total PLHIV with AIDS ilness *A*, and **(E–H)** for control variables under the scenario when condom use and case detection implemented together.

The last simulation was conducted to assess the impact of combining condom usage and case detection in reducing the spread of HIV/AIDS in Eswatini. The results are presented in Figure 19, with the dynamics of *u*_1_ and *u*_2_ shown in panels (E–H), respectively. We observe that the intervention involving condom use should be initiated at a high rate at the beginning of the simulation while the case detection followed the dynamic of infected individuals. As a result, we witness an initial increase in the number of susceptible individuals [panel (A)] and a significant decrease in the PLHIV without treatment and PLHIV with AIDS illness [panels (B, D), respectively]. On the other hand, since the number of infected PLHIV without treatment is already reduced due to condom use, then the number of treated PLHIV is not significantly different in the case of no control scenario. Finally, the combination of condom use and case detection leads to a significant reduction in the number of PLHIV. The total number of infections averted using this strategy is 7.55 × 10^6^ at an optimal cost of 9.81 × 10^14^.

### Cost-effectiveness analysis

In this section, we will calculate the most effective strategy between strategies 1,2, and 3 based on its average cost-effectiveness ratio (ACER) values. Several assumptions need to be elucidated in the cost-effectiveness calculations in this section. In computing the total cost (*TC*), it is assumed that the total expenses incurred as a consequence of heightened control interventions constitute the overall cost. On the other hand, total infections averted (*TIA*) are derived from the total number of individuals successfully prevented from infection as a consequence of control interventions. For further clarity, refer to Equations (8) and (9) below.


(8) $$\begin{array}{l} \text{ACER}=\frac{\text{Total cost for intervention (TC)}}{\text{Total number of infections averted (TIA)}}, \end{array}$$

where


(9) $$\begin{array}{ll} \text{TC} & =\int_{0}^{t_{f}}[\omega_{1}u_{1}(I_{1}^{‡}+I_{2}^{‡}+I_{3}^{‡}+T_{1}^{‡}+T_{2}^{‡}+T_{3}^{‡})+\omega_{2}u_{2}I_{1}^{‡}+\\ \; & \omega_{3}I_{2}^{‡}+\omega_{4}I_{3}^{‡}]dt\;\text{and}\\ \text{TIA} & =\int_{0}^{t_{f}}\left[\sum_{i=1}^{3}\left(I_{i}^{†}-I_{i}^{‡}\right)+\sum_{i=1}^{3}\left(T_{i}^{†}-T_{i}^{‡}\right)\right]dt, \end{array}$$

where symbol † and ‡ represent simulation results, without and with control, respectively. A smaller value of ACER represents the most effective strategy. The results of TC, TIA, and ACER are given in Table 2.

**Table 2:** Simulation result for cost-effectiveness analysis.

**Scenario**	**TC**	**TIA**	**ACER**
1 (*u*_1_ ≠ 0, *u*_2_ = 0)	2.06 × 10^11^	7.59 × 10^6^	2.71 × 10^4^
2 (*u*_1_ = 0, *u*_2_ ≠ 0)	2.52 × 10^16^	3.74 × 10^4^	6.74 × 10^11^
3 (*u*_1_ ≠ 0, *u*_2_ ≠ 0)	9.81 × 10^14^	7.55 × 10^6^	1.29 × 10^8^

**Figure 20:**
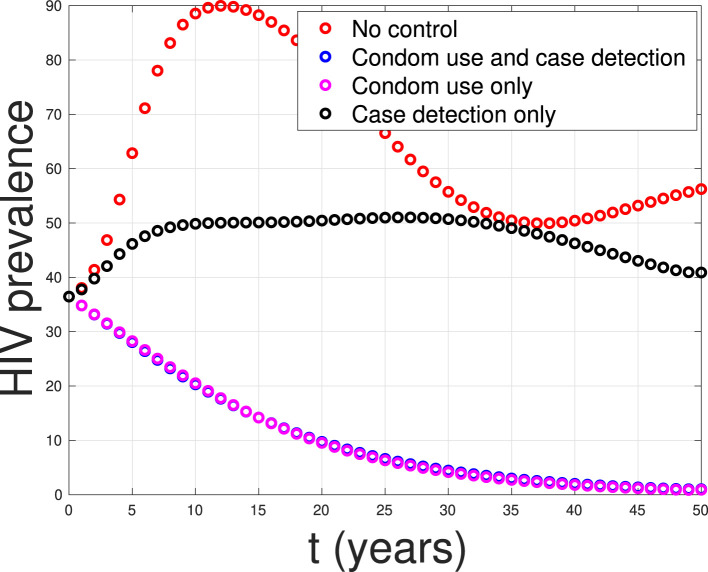
A comparison between control scenarios on the HIV prevalence in Eswatini.

Based on the calculations above, we can conclude that strategy 1, which focuses solely on condom use as the single intervention, is the most effective strategy. It is followed by strategy 3, which combines condom use and case detection. Strategy 2, which relies solely on case detection as the single intervention, is the least effective strategy. Figure 20 shows the impact of all possible scenarios on HIV prevalence in Eswatini. We can observe that the HIV prevalence between scenario 1 (condom use only) and scenario 3 (condom use and case detection) is only slightly different. This affirms that case detection is less effective in reducing HIV prevalence when the implementation of condom use is already taking place at maximum effort.

## 5 Conclusion

In this study, we developed a mathematical model to assess the effectiveness and cost-effectiveness of various strategies aimed at controlling the spread of HIV/AIDS. We considered the impact of case detection, treatment, and condom use in our model. One key aspect of our model is its acknowledgment of the fact that treatment outcomes may not always be successful, making it a more realistic representation of the disease's dynamics. We begin our analysis for a special case model, where we do not consider the number of CD4+T cells. Our mathematical investigation on the expression of the equilibrium points and the reproduction number reveals the potential of case detection to reduce the reproduction number of the virus.

Our comprehensive analysis is extended to the complete model, where we assessed the stability of the HIV/AIDS-free equilibrium point. We found that this equilibrium is stable when the control reproduction number is less than one, indicating the feasibility of disease containment.

To validate our model, we estimate our parameter values using data from four different countries: Eswatini, Lesotho, Botswana, and South Africa. Parameter estimation was performed using incidence data from these regions, and we found that the reproduction of each country is larger than one, which indicates the endemicity of HIV/AIDS in those countries. Furthermore, sensitivity analysis sheds light on the impact of condom use and case detection on HIV spread dynamics. It allowed us to identify the most influential factors in disease control.

Finally, our study delved into optimal control strategies, considering the dynamics of infected individuals when all control variables are time-dependent. From a cost-effectiveness perspective, we found that employing condom use as a sole intervention is the most effective strategy in terms of the average cost for each averted infected individual. It is worth noting that condom use not only serves as a cost-effective approach but is also a crucial tool in preventing the transmission of HIV/AIDS. While the simulation results for case detection in this research indicate its lesser effectiveness in reducing the number of HIV prevalence, it still holds importance. Case detection, despite its limitations, plays a crucial role in assisting the government in mitigating the broad social impact of HIV in the population. In light of the complexities surrounding HIV/AIDS, a comprehensive strategy that combines both condom use and case detection could offer a more robust approach to tackling the multifaceted challenges posed by the HIV epidemic. In conclusion, our research provides valuable insights into the control of HIV/AIDS, offering a comprehensive assessment of strategies and emphasizing the importance of case detection as a highly efficient and cost-effective means of disease containment.

Beyond its potential in reducing HIV spreads, there are several issues about the implementation of condom use and case detection. In societies where discussions about sex and sexual health are taboo or stigmatized, condom use campaigns may face challenges in gaining acceptance. Cultural norms and religious beliefs can influence attitudes toward condom use. Accessibility to condoms may be limited in certain cultural contexts due to factors such as affordability, availability, and distribution channels. Addressing these barriers is crucial for the success of condom use campaigns. On the other hand, HIV-related stigma and discrimination can impede case detection efforts. Fear of being ostracized or discriminated against may discourage individuals from seeking HIV testing and treatment. Privacy concerns are paramount in HIV testing and case detection. Trust in healthcare systems is essential for successful case detection. In some communities, historical distrust or negative experiences with healthcare providers may affect the willingness to engage with HIV testing and treatment services. In summary, the effectiveness of condom use campaigns and case detection for HIV depends on their alignment with social and cultural contexts. Interventions need to be culturally sensitive, addressing barriers related to stigma, discrimination, and accessibility to effectively prevent the spread of HIV and promote early detection and treatment. In diverse social and cultural settings, collaborating with local communities, leaders, and organizations can enhance the relevance and acceptance of these interventions.

Although the research results in this article show in-depth insights into the potential use of condoms and early detection in reducing HIV prevalence in case studies across four countries, there are still some aspects that can be further developed to achieve more satisfying outcomes. One of these aspects is the fact that condom use is not only for suppressing or preventing the spread of HIV but also for other sexually transmitted infections (STIs) such as syphilis, chlamydia, herpes, and human papillomavirus (HPV). Therefore, condom use as an intervention can be applied in models that consider co-infection between two or more STIs. Only few researchers have discussed coinfection models involving HIV and other STIs, as seen in (47–51). The use of condoms as an intervention that can prevent both diseases simultaneously would be intriguing to explore in future research.

## Data availability statement

The original contributions presented in the study are included in the article/Supplementary material, further inquiries can be directed to the corresponding author.

## Author contributions

DA: Conceptualization, Formal analysis, Investigation, Methodology, Supervision, Validation, Visualization, Writing – original draft, Writing – review & editing. RD: Formal analysis, Investigation, Writing – original draft. SK: Methodology, Software, Validation, Visualization, Writing – review & editing. JW: Data curation, Investigation, Software, Validation, Visualization, Writing – review & editing. PK: Formal analysis, Funding acquisition, Investigation, Supervision, Validation, Writing – review & editing. MS: Investigation, Software, Visualization, Writing – review & editing.

## Footnotes

^1^AIDS by the Numbers (2023). Available online at: https://www.unaids.org/en.

^2^The Global HIV and AIDS Epidemic (2023). Available online at: https://www.hiv.gov/hiv-basics/overview/data-and-trends/global-statistics/#::text=HIV.
