# Supplementary material for: Understanding HIV/AIDS dynamics: insights from CD4+T cells, antiretroviral treatment, and country-specific analysis
Source: Front Public Health. 2024 Apr 11;12:1324858. doi: 10.3389/fpubh.2024.1324858 (PMC11043473; doi:10.3389/fpubh.2024.1324858)
Supplement: Supplementary file 1 [file Table_1.pdf]

Understanding HIV/AIDS Dynamics: Insights from CD4+T Cell, Antiretroviral Treatment, and Country-Specific Analysis

Dipo Aldila [1,*], Ranandha P. Dhanendra [1] andSarbaz H. A. Khoshnaw [2] Juni Wijayanti Puspita[3] Putri Zahra Kamalia [1] Muhammad Shahzad[4]

[1] *Department of Mathematics, Universitas Indonesia, Depok 16424, Indonesia.*
[2] *Department of Mathematics, University of Raparin, Ranya 46012, Kurdistan Region of Iraq.*
[3] *Department of Mathematics, Universitas Tadulako, Palu 94118, Indonesia.*
[4] *Department of Mathematics & Statistics, The University of Haripur, KP, Haripur, 22620, Pakistan*

Correspondence*:
D. Aldila
aldiladipo@sci.ui.ac.id

1 APPENDIX 1: HIV/AIDS PREVALENCE DATA FROM ESWATINI, LESOTHO, BOTSWANA, AND SOUTH AFRICA FROM 1990 TO 2021.

Country:	Eswatini															
Year:	1990	1991	1992	1993	1994	1995	1996	1997	1998	1999	2000	2001	2002	2003	2004	2005
Prev.:	1.1	2.1	3.8	6.3	9.3	12.5	15.5	18	20	21.5	22.7	23.6	24.3	25	25.5	25.9
Year:	2006	2007	2008	2009	2010	2011	2012	2013	2014	2015	2016	2017	2018	2019	2020	2021
Prev.:	26.3	26.7	27.1	27.5	27.9	28.2	28.5	29	29.4	29.6	29.8	29.7	29.4	29.1	28.6	27.9

Country:	Lesotho															
Year:	1990	1991	1992	1993	1994	1995	1996	1997	1998	1999	2000	2001	2002	2003	2004	2005
Prev.:	1.8	2.9	4.6	6.9	9.6	12.5	15.2	17.4	19.2	20.5	21.5	22.1	22.5	22.7	22.9	23.1
Year:	2006	2007	2008	2009	2010	2011	2012	2013	2014	2015	2016	2017	2018	2019	2020	2021
Prev.:	23.3	23.5	23.7	24	24.2	24.4	24.6	24.8	24.7	24.6	24.2	23.6	23	22.3	21.6	20.9

Country:	Botswana															
Year:	1990	1991	1992	1993	1994	1995	1996	1997	1998	1999	2000	2001	2002	2003	2004	2005
Prev.:	6.4	8.8	11.5	14.3	17.1	19.6	21.7	23.4	24.6	25.4	25.8	25.9	25.7	25.4	25	24.6
Year:	2006	2007	2008	2009	2010	2011	2012	2013	2014	2015	2016	2017	2018	2019	2020	2021
Prev.:	24.3	24	23.7	23.5	23.2	23	22.7	22.4	22	21.7	21.3	20.9	20.4	19.8	19.2	18.6

Country:	South Africa															
Year:	1990	1991	1992	1993	1994	1995	1996	1997	1998	1999	2000	2001	2002	2003	2004	2005
Prev.:	0.8	1.4	2.3	3	4	5.2	6.5	7.9	9.1	10.3	11.3	12.3	13	13.7	14.1	14.6
Year:	2006	2007	2008	2009	2010	2011	2012	2013	2014	2015	2016	2017	2018	2019	2020	2021
Prev.:	14.9	15.2	15.6	15.9	16.3	16.8	17.2	17.5	17.9	18.2	18.4	18.5	18.6	18.6	18.4	18.3

Table 1. HIV prevalence data from Eswatini, Lesotho, Botswana, and South Africa [51].

2 APPENDIX 2: ESTIMATION RESULTS

Country	Estimated Initial Condition		Estimated Parameter	$\mathcal{R}_0$
Eswatini	$S(0) = 669026$;	$I_1(0) = 10000$;	$\beta_u = 1.4888 \times 10^{-6}$; $\beta_t = 1.4888 \times 10^{-7}$;	1.095
	$I_2(0) = 1001$;	$I_3(0) = 12198$;	$\tau_1 = 0.6546$; $\tau_2 = 0.8870$; $\tau_3 = 2.6261$;	
	$T_1(0) = 10000$;	$T_2(0) = 10000$;	$\kappa = 0.3918$; $\eta = 0.2222$	
	$T_3(0) = 2000$;	$A(0) = 1000$		
Lesotho	$S(0) = 1262898$;	$I_1(0) = 20000$;	$\beta_u = 8.1185 \times 10^{-7}$; $\beta_t = 8.1199 \times 10^{-8}$;	1.682
	$I_2(0) = 20000$;	$I_3(0) = 10000$;	$\tau_1 = 0.5$; $\tau_2 = 0.5$; $\tau_3 = 0.7833$;	
	$T_1(0) = 20000$;	$T_2(0) = 5000$;	$\kappa = 0.5359$; $\eta = 0.3997$	
	$T_3(0) = 2000$;	$A(0) = 1000$		
Botswana	$S(0) = 1341304$;	$I_1(0) = 20000$;	$\beta_u = 7.4678 \times 10^{-7}$; $\beta_t = 8.0531 \times 10^{-8}$;	1.732
	$I_2(0) = 1000$;	$I_3(0) = 50000$;	$\tau_1 = 0.5355$; $\tau_2 = 0.5355$; $\tau_3 = 0.5386$;	
	$T_1(0) = 40000$;	$T_2(0) = 60000$;	$\kappa = 0.5733$; $\eta = 0.3999$	
	$T_3(0) = 20000$;	$A(0) = 1000$		
South Africa	$S(0) = 36540015$;	$I_1(0) = 250000$;	$\beta_u = 3.3870 \times 10^{-8}$; $\beta_t = 3.3926 \times 10^{-9}$;	1.65
	$I_2(0) = 189234$;	$I_3(0) = 192419$;	$\tau_1 = 0.7873$; $\tau_2 = 0.8844$; $\tau_3 = 1.3124$;	
	$T_1(0) = 69426$;	$T_2(0) = 98171$;	$\kappa = 0.5273$; $\eta = 0.3335$	
	$T_3(0) = 166823$;	$A(0) = 13887$		

Table 2. Estimation results for the best-fit parameters of the proposed HIV Model.
